# Comparative Genomics of Interreplichore Translocations in Bacteria: A Measure of Chromosome Topology?

**DOI:** 10.1534/g3.116.028274

**Published:** 2016-03-30

**Authors:** Supriya Khedkar, Aswin Sai Narain Seshasayee

**Affiliations:** * National Centre for Biological Sciences, GKVK, Bellary Road, Bangalore 560065, Karnataka, India; † SASTRA University, Thanjavur 613401, Tamil Nadu, India

**Keywords:** genome evolution, genome architecture, chromosome topology, gene order conservation

## Abstract

Genomes evolve not only in base sequence but also in terms of their architecture, defined by gene organization and chromosome topology. Whereas genome sequence data inform us about the changes in base sequences for a large variety of organisms, the study of chromosome topology is restricted to a few model organisms studied using microscopy and chromosome conformation capture techniques. Here, we exploit whole genome sequence data to study the link between gene organization and chromosome topology in bacteria. Using comparative genomics across ∼250 pairs of closely related bacteria we show that: (a) many organisms show a high degree of interreplichore translocations throughout the chromosome and not limited to the inversion-prone terminus (*ter*) or the origin of replication (*oriC*); (b) translocation maps may reflect chromosome topologies; and (c) symmetric interreplichore translocations do not disrupt the distance of a gene from *oriC* or affect gene expression states or strand biases in gene densities. In summary, we suggest that translocation maps might be a first line in defining a gross chromosome topology given a pair of closely related genome sequences.

Chromosome compaction is a necessary feature of all living cells including bacteria. It is intricately linked to chromosome topology and the selective pressures introduced by gene organization along the genome sequence.

In many bacteria, nucleoid-associated proteins (Dillon and Dorman 2010) and topoisomerases (DiNardo *et al.* 1982; Pruss *et al.* 1982) establish local DNA geometries, which include bending (Stella *et al.* 2010), bridging (Dame *et al.* 2006; Dupaigne *et al.* 2012; Liu *et al.* 2010; Maurer *et al.* 2009), wrapping, and relative over-/underwinding of DNA. These not only help compact DNA, but also play a role in constraining replication (Gruber *et al.* 2014; X. Wang *et al.* 2014a), recombination (Esposito and Gerard 2003; Shanado *et al.* 2001), and transcription (Browning *et al.* 2010; Dillon and Dorman 2010).

Zooming out, two broad chromosome topologies have been discerned across model bacteria. First, in *Escherichia coli*, genetic, recombination-based contact maps of the chromosome showed the presence of four macrodomains, defined by the preferential occurrence of recombination events within rather than across these macrodomains (Valens *et al.* 2004). Chromosome topology in *E. coli* is further characterized by the localization of the origin of replication (*oriC*) at midcell and the transverse spread of the left and right replichores in opposite directions toward the cell poles (Nielsen *et al.* 2006; Wang *et al.* 2006). This is referred to as the “*transverse*” chromosome topology.

On the other hand, high-resolution 5C and Hi-C chromosome contact maps of *Caulobacter crescentus* present a different picture by revealing a “*longitudinal*” topology (Le *et al.* 2013; Umbarger *et al.* 2011). In the *C. crescentus* chromosome, symmetric contacts (defined as contacts between loci equidistant from *oriC*) between the two replichores (two arms of the chromosome extending from *oriC* at one pole to the terminus at the other pole) could be discerned, possibly resulting in a helical structure (Le and Laub 2014); such contacts are rare in the *E. coli* chromosome (Nielsen *et al.* 2006; Wang *et al.* 2006). Similarly, recent microscopy experiments showed that the two replichores of *Mycobacterium smegmatis* colocalize throughout replication (Santi and McKinney 2015). For both the topologies, distinguished by the presence or absence of close interreplichore contacts, it is apparent that *oriC* and the terminus (*ter)* rarely contact each other. Finally, the chromosome of *Bacillus subtilis* has been shown to oscillate between these two conformations (X. Wang *et al.* 2014b).

The molecular mechanisms and players underlying the establishment of a defined chromosome topology are only beginning to be understood. These include DNA-binding proteins such as MatP (Mercier *et al.* 2008), the SMC complex (Le *et al.* 2013), nucleoid-associated proteins (Umbarger *et al.* 2011; Wang *et al.* 2011), and noncoding RNAs (Qian *et al.* 2015). Chromosome topologies might also be influenced by active cellular processes such as DNA replication and transcription. In *E. coli*, replication has been implicated in shaping the topology of the chromosome (Cagliero *et al.* 2013). Modeling of the bacterial chromosome using tools from polymer science has indicated that large switches in chromosome topology can occur by the mere repositioning of *oriC* or *ter* sites within the cell (Junier *et al.* 2014). In *C. crescentus*, highly expressed genes act as topological domain boundaries on the chromosome (Le *et al.* 2013).

An important question then, is whether chromosome topology has any functional relevance. In other words, is chromosome topology under evolutionary selection? Whereas local DNA geometries, including the writhe of DNA around a nucleoid-associated protein, has definite effects on transcription and DNA repair (Dillon and Dorman 2010; Dorman 2013), whether the overall shape of the chromosome has any function, beyond being a solution for compacting DNA within the confines of a cell, is unclear. Long-range interactions between chromosomal segments might enable coexpression of genes encoded in these segments (Wang *et al.* 2016). The collapse of transcriptionally silent genes by the global repressor H-NS into a few foci in *E. coli* (Wang *et al.* 2011) – akin to the spatial clustering of distinct heterochromatin sequences in the eukaryotic nucleus − indicates conservation of this topological characteristic across phyla; whether this is a requirement for transcriptional silencing remains an open question. This point, however, must be tempered by the fact that fluorescent tags used for observing the foci may actually be the cause of some of the clustering effects (S. Wang *et al.* 2014). Further, Esnault *et al.* (2007) have revealed that the effects of DNA inversions on growth are dependent on their impact on chromosome topology; *i.e.*, inversions that span multiple topological macrodomains of the chromosome are more detrimental than those that are limited to a single domain, thus suggesting that chromosome topology might be under evolutionary selection.

While the study of aspects of bacterial chromosome topology and of its evolution is still nascent (Lagomarsino *et al.* 2015), we have a substantially deeper understanding of chromosome architecture in terms of how genes are organized on the bacterial chromosome (Rocha 2008). Depending on the time spent per cell cycle by a bacterial cell in replicating the genome, there is a clear difference in gene dosage between *oriC* and *ter* (Block *et al.* 2012; Schmid and Roth 1987). This is particularly apparent in fast-growing bacteria like *E. coli*, where *oriC* fires multiple times per cell cycle (Cooper and Helmstetter 1968). As a potential consequence of this critical difference in gene dosage between *oriC* and *ter*, essential and highly expressed genes are encoded close to *oriC* (Rocha 2004). In contrast, many stress-responsive (Sobetzko *et al.* 2012) and horizontally acquired genes are encoded around *ter* (Zarei *et al.* 2013). Finally, a majority of bacterial genes – particularly those that are essential – are encoded on the leading strand (Rocha and Danchin 2003), consistent with the idea that head-on collision between the two polymerases is mutagenic and detrimental (Mirkin and Mirkin 2005).

Gene organization changes as species diverge (Tamames 2001). For example, comparative genomics has shown that loci immediately adjacent to *oriC* and *ter* are prone to interreplichore inversions (Eisen *et al.* 2000; Suyama and Bork 2001). Previous anecdotal genome alignment analyses have also revealed the presence of interreplichore translocations in certain pairs of genomes; more so for distantly related organisms. These result in the classical X-shaped pattern in sequence comparison dot plots (Eisen *et al.* 2000; Koonin and Wolf 2008; Mira *et al.* 2002; Suyama and Bork 2001; Tillier and Collins 2000).

Here we interrogate the relationship between chromosome topology and gene organization across bacteria. Specifically, we ask whether we can exploit the wealth of genome sequence data to explore the utility of gene organization as a tool to infer the topology of a bacterial chromosome, thus accounting in part for the paucity of chromosome conformation data across bacteria.

## Materials and Methods

### Data

We obtained genomic DNA [.fna files], RNA [.frn;.rnt files], and protein [.faa] sequence data for ∼3000 completely sequenced bacteria from the National Center for Biotechnology Information (NCBI). We obtained gene coordinates, protein IDs, and Cluster of Orthologous genes (COG) annotations from .ptt files (ftp://ftp.ncbi.nlm.nih.gov/genomes/archive/old_refseq/Bacteria/).

### R-factor calculation

We calculated R-factor (*R*_f_) as a physiologically relevant measure of replication-dependent gene dosage gradient down the *oriC−ter* axis. This considers both replication time and minimum doubling time. It was first described by Couturier and Rocha (2006).

The R-factor (*R*_f_) was defined as ***R*_f_** = ***T*_R_**/***T*_D,_** where ***T*_R_** stands for the estimated time taken for a full round of chromosomal replication, and ***T*_D_** represents the minimum doubling time. ***T*_R_** was defined as the ratio of half the genome size and an average speed of DNA replication *in vivo*, which was taken to be 600 nt/sec (Reyes-Lamothe *et al.* 2008) for all bacteria. This is an oversimplification: DNA replication speeds vary across bacteria, but such data are not readily available, and the extent of its variability is unknown. ***T*_D_** was obtained for ∼100 bacteria from Freilich *et al.* (2009).

### Distance calculation

We determined the location of a gene in the genome as its distance from *oriC* down the replichore. We first obtained *oriC* and *ter* coordinates for 1528 completely sequenced bacteria from DoriC (Gao and Zhang 2007). We calculated the shortest distance of all genes from the origin of replication and divided it by half the genome size. The normalized distances of genes from *oriC* varied from 0 to 1, 0 being close to *oriC* and 1 being close to *ter*.

### Dataset for pairwise comparisons

We extracted a 16S rRNA sequence for 1528 completely sequenced bacteria. For organisms with multiple 16S rRNAs, we obtained the sequence of the copy closest to *oriC*. We did a 16S rRNA sequence similarity search using the Needleman−Wunsch global alignment algorithm (Needleman and Wunsch 1970), implemented in EMBOSS (EMBOSS:6.4.0.0) (Rice *et al.* 2000) using default parameters. We then considered all pairs of bacteria with greater than 97% 16S rRNA sequence identity. For further analysis we included only those pairs of genomes encoding the same number of 16S rRNA genes, as determined from the .rnt files in NCBI. This ensures that pairs of genomes used for comparative genomics would have similar growth rates. As described in *Results*, we removed pairs involving: (a) strains of the same species; (b) redundancy; and (c) obligate parasites. Our final dataset consisted of 232 individual bacterial species forming 262 pairs of bacteria with the same 16S rRNA copy number and ≥97% 16S rRNA sequence identity.

### Determination of orthologs

For the 262 pairs of bacteria, using the protein sequences (data from .faa files) we did an all *vs.* all bidirectional best-hit *phmmer* (HMMER 3.0, March 2010) search (Finn *et al.* 2011) with default parameters and an E-value cut off of 10^−10^. The data pertaining to all the identified orthologs used in this study for 262 pairs of bacteria can be accessed here: http://bugbears.ncbs.res.in/genome_architecture/.

### Correction for the effect of phylogenetic distance

The correction for the dependence of various measures of gene organization on phylogenetic distance (mentioned in the main text) was implemented as follows. We fitted the dependent variable (*y*) against 16S sequence similarity (*x*) using local polynomial regression (LOESS). The residual of this fit was calculated by subtracting individual values of *y* from the predicted values of *y* obtained after the LOESS regression. These residual values represented the component not explained by phylogenetic distance.

### Assignment of chromosomal bins

We defined 25% of the chromosome centered around *oriC* as the O or origin bin. Twenty-five percent of the chromosome immediately to the right (along the text of the genome sequence) and to the left of the O bin was termed R and L, respectively. The remaining 25% of the chromosome, which includes *ter*, was termed the T bin.

### Determination of functional enrichment

Functional annotations for individual orthologs were extracted from NCBI [.ptt files]. These COG annotations were used to determine enrichment of specific functions in each bin. Twenty-three COG classes (Supplemental Material, File S1) belonging to three broad functions − information storage and processing, cellular process, and signaling and metabolism – were considered. For each genome, Fisher’s exact test was used to determine statistical significance for the enrichment of each COG class in any given bin. The obtained *P*-values were then corrected for multiple comparisons using Bonferroni correction and were negative log base 10 transformed.

### Prediction of horizontally acquired genes

We used Alien Hunter (Release 1.7) (Vernikos and Parkhill 2006) with default parameter values to predict horizontally acquired genes. For this analysis, bacteria with guanine and cytosine (GC) content in the range of 40–60% were considered. The coordinates of predicted horizontally acquired genes obtained from Alien Hunter were further used to assign them to individual chromosomal bins. Depletion of predicted horizontally acquired genes in an individual bin was determined using Fisher’s exact test adjusted for multiple testing by Bonferroni.

### Analysis of C. crescentus 5C data

We obtained chromosome conformation capture data for *C. crescentus* NC_011916 from Le *et al.* (2013)(Gene Expression Omnibus − accession no. GSE45966; sample GSM1120445). From this dataset we extracted normalized contact frequency information for all bins showing interreplichore contacts.

Ortholog positions that showed interreplichore translocations in *C. crescentus* NC_011916 and its closely related bacterial pairs were pooled together. For all these translocated loci we extracted the contact frequency information from the above dataset. This formed the test dataset consisting of contact frequencies for interreplichore translocated loci. The control set included all other pairs of interreplichore loci that did not show translocations. The two distributions were compared using the Wilcoxon test.

### Phylogenetic tree

The 16S rRNA sequence of the rRNA copy closest to origin of replication was obtained for all 232 bacterial species used in this study. 16S rRNA multiple sequence alignment was generated using MUSCLE (Edgar 2004), as implemented in MEGA version 6 (Tamura *et al.* 2013) using default parameters. A maximum likelihood (ML) tree was generated in MEGA version 6 using all sites in the 16S rRNA alignment. The Tamura and Nei (1993) nucleotide substitution model was used to infer the tree. The phylogenetic tree was visualized using iTOL (Letunic and Bork 2011), and growth rate and interreplichore translocation frequency were overlaid on the phylogenetic tree.

### Randomization protocol

For each pair of bacteria showing interreplichore translocations, the assignment of an ortholog to a gene was randomized, thus creating random pairing between genes. Then *D*_inter_ was calculated for each gene pair in this randomized dataset. These randomized datasets were used to compute a null distribution for *D*_inter_ in Figure 4B and Figure 5A, and as the null expectation for the frequency of interreplichore strand flips in Figure 5B.

All the statistical tests and data visualizations were performed using *R* (unless specified otherwise).

### Data availability

The authors state that all data necessary for confirming the conclusions presented in the article are represented fully within the article.

## Results

### A comparative genomic analysis of gene position conservation

In order to infer chromosome topologies and their association with gene dosage gradients down the *oriC*−*ter* axis, we established a comparative genomic framework. Our approach is based on the frequencies of translocations between distal segments of the chromosome, as defined by sequence comparisons between pairs of closely related organisms. This is based on the supposition that spatially proximal regions of the chromosome would recombine more frequently, and recombination combined with selection would result in the establishment of translocation events between such loci. This approach is similar to the experimental mapping of the topology of the *E. coli* chromosome using recombination frequencies between distal loci (Valens *et al.* 2004), with the difference that the translocations we identify are products of recombination followed by selection and probably drift over long timescales.

We obtained complete genome sequences and gene annotations for ∼3000 fully sequenced bacterial genomes from the NCBI. Because gene dosage gradients down the *oriC*−*ter* axis are dependent on growth rates, we sought to annotate each organism in our collection of genomes with growth rate information. To do so, we assembled a dataset of growth rates in the form of minimum doubling times for over 100 bacteria from Freilich *et al.* (2009).

**Figure 1:**
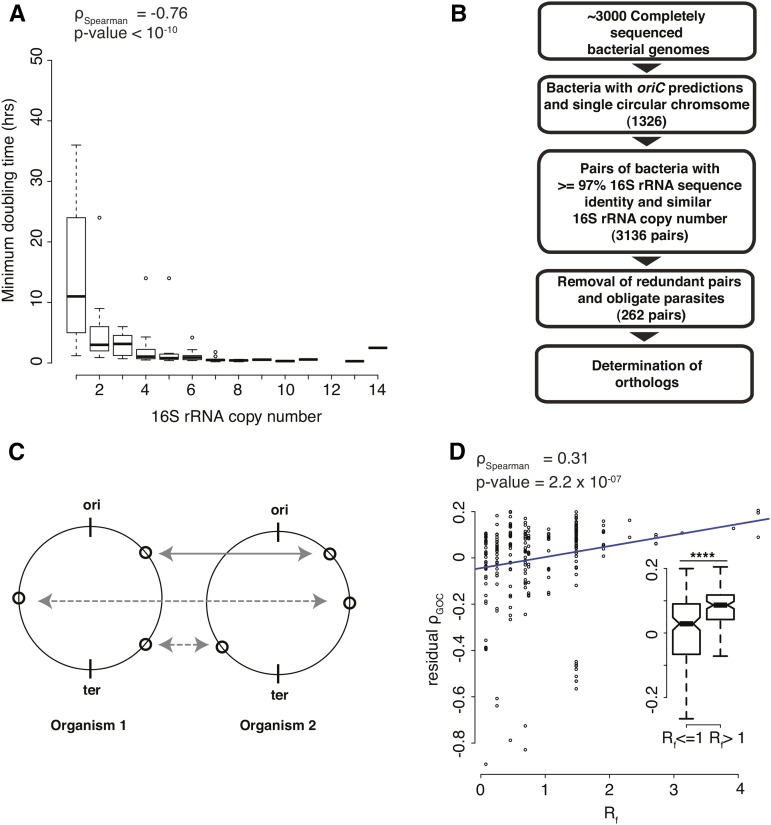
Comparative Genomics Framework. (A) Plot representing the relationship between minimum doubling time (hr) and 16S rRNA copy number (ρ_Spearman_ = −0.76, *P*-value <10^−10^). Minimum doubling time values >50 hr are not represented in the plot. (B) Flowchart showing the selection of 262 pairs of bacteria used in this study. (C) Schematic showing the conservation of gene position (solid lines) and gene translocations (dashed lines) between pairs of bacteria. (D) Plot representing the correlation between residual ρ_GOC_ and *R*_f_ (ρ_Spearman_ = 0.31, *P*-value = 2.2 × 10^−7^). Inner panel shows a statistically significant difference between residual ρ_GOC_ for slow (*R*_f_ ≤1) and fast (*R*_f_ > 1) growing bacteria (*P*-value = 3.3 × 10^−6^, Wilcoxon test). Asterisks indicate *P*-value < 10^−3^.

Although growth rate information is sparse relative to genome sequence data, we know that the number of rRNA genes encoded by a bacterium is a predictor of its growth rate (Condon *et al.* 1995; Klappenbach *et al.* 2000), with fast-growing bacteria encoding more rRNA operons than slow-growing ones. Consistent with this knowledge, we observed a negative correlation (ρ_Spearman_= −0.76) between minimum doubling times and rRNA copy numbers for these ∼100 bacteria (Figure 1A).

Based on the fit between minimum doubling times and rRNA copy numbers, we estimated the growth rate expected for each rRNA copy number observed in our dataset. However, growth rate itself is not necessarily a strong predictor of differences in gene dosage between *oriC*-proximal and *ter*-proximal loci. Instead, a better correlate is the *R*-factor (*R*_f_), defined as the number of replication cycles per cell division. Using a previous study from the Rocha group as a guide (Couturier and Rocha 2006), we calculated the *R*_f_ for each bacterium as described in *Materials and Methods*. We then defined organisms that are expected to initiate replication more than once per cell cycle on average (*R*_f_ > 1) as fast-growing, and the rest as slow-growing. We also estimated *R*-factor independently using a larger, more recent dataset (Vieira-Silva and Rocha 2010) of minimum doubling times for ∼200 bacteria, and found estimates of R-factor derived from this dataset to be consistent with those used in this study (Figure S1).

We organized the ∼3000 genomes into 262 genome pairs on the basis of phylogenetic distance and growth rate. We considered pairs of bacteria with ≥97% 16S rRNA sequence identity (Figure S2) and selected those with similar growth rates (same 16S rRNA copy numbers) (Figure 1B). We then removed: (a) pairs of strains of the same species, which typically showed very few long-range translocations (Figure S3); (b) redundant pairs to minimize overrepresentation of certain phylogenetic groups: for example, there are several hundred comparisons involving *E. coli* and *Salmonella*, and of these one was picked at random; and (c) pairs of obligate parasites with genomes smaller than 1.3Mb (Koonin and Wolf 2008), whose reduced genomes are known to be stable with exceptionally high gene order conservation (Tamas 2002; Mira *et al.* 2002). Thus, our final selection consisted of 232 individual bacterial species forming 262 pairs of bacteria with the same 16S rRNA copy number and ≥97% 16S rRNA sequence identity. A phylogenetic tree of these bacteria, based on their 16S rRNA sequences, is shown in Figure S4.

For all the 262 pairs of bacteria, we identified gene orthologs by bidirectional best-hit phmmer (E-value <10^−10^). We found that ∼72% (median) of genes in one organism had an ortholog in its partner genome. The percentage of genes conserved in a pair of organisms decreased with phylogenetic distance between the compared genomes and showed little correlation with growth rate (Figure S5).

For all these genes, we computed the position of each gene on its chromosome. The position of a gene was defined by its distance from *oriC* along the path of the replication fork and normalized to genome size. Positions equidistant from *oriC* on either replichore were considered equivalent in these calculations. Thus, a difference in gene position would indicate a difference in dosage, depending on the organism’s R-factor. For each genome and its partner, we defined gene position vectors, in which each gene with an identifiable ortholog in its partner genome was represented by its distance from *oriC*. Then, for each genome pair, we computed the correlation coefficient between their gene position vectors (Figure 1C) and treated this correlation as an estimate of gene order conservation (ρ_GOC_).

As expected for closely related organisms, the average gene order conservation across the 262 pairs of organisms studied here was high (median ρ_GOC_ ∼0.9). Loss of gene order conservation is dependent on phylogenetic distance (Suyama and Bork 2001; Tamames 2001). Hence, we calculated residual ρ_GOC_ after correcting for the dependence of ρ_GOC_ on phylogenetic distance (Figure S6). We found that the residual ρ_GOC_ increases with increasing R-factor (*R*_f_) weakly but in a statistically significant fashion (*P*-value= 3.3 × 10^−6^, Wilcoxon test; Figure 1D), consistent with earlier calculations by Rocha and colleagues (Couturier and Rocha 2006).

**Figure 2:**
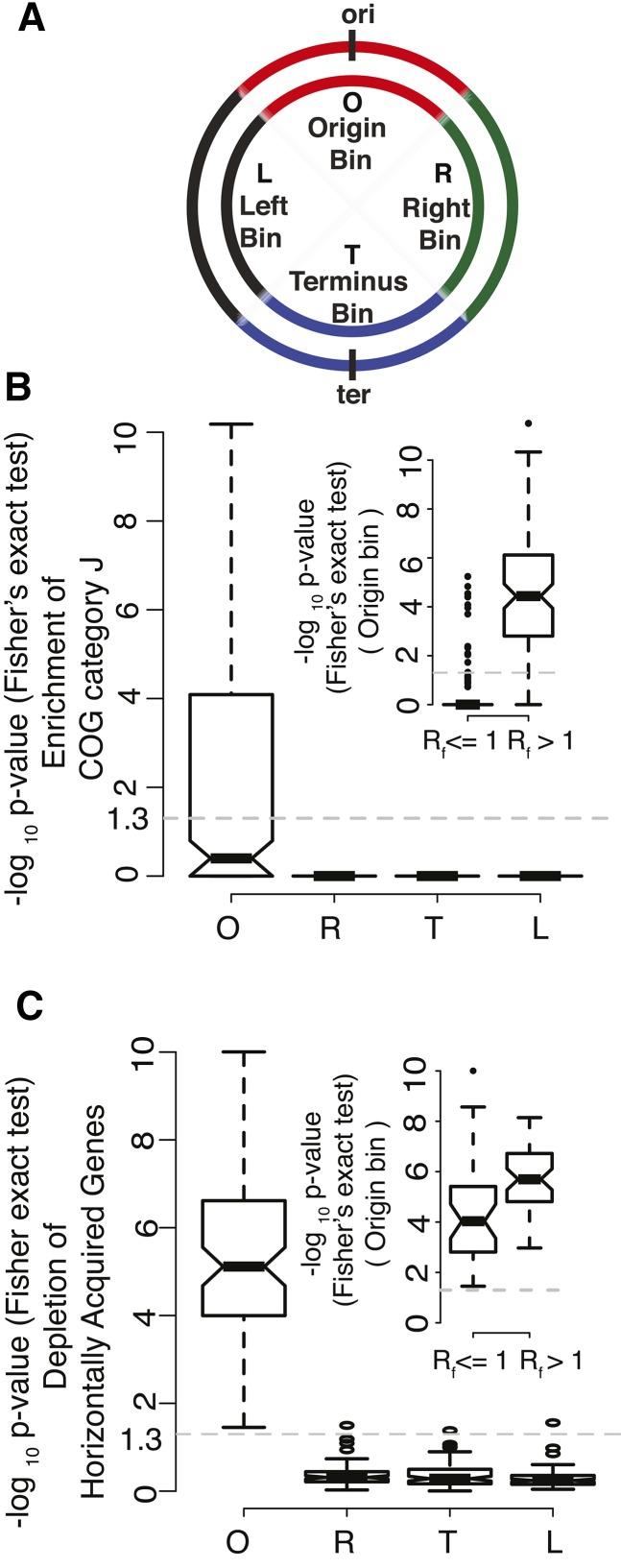
Chromosomal bin-based conservation of function. (A) Schematic representing equal sized bacterial chromosomal segments. Red: Origin bin (O); green: Right bin (R); blue: Terminus bin (T); black: Left bin (L). (B) Boxplot showing the enrichment of translation and ribosome biogenesis genes (COG category J) in O, R, T, and L chromosomal bins. (The dashed gray line at *y* = 1.3 corresponds to a *P*-value of 0.05. Values ≥1.3 are statistically significant.). Inner panel shows the enrichment of COG J in the O bin of fast-growing bacteria (*R*_f_ > 1). (C) Boxplot showing the depletion of horizontally acquired genes in different chromosomal bins. Inner panel showing the depletion of horizontally acquired genes in the O bin of both slow- (*R*_f_ ≤ 1) and fast (*R*_f_ > 1) growing bacteria.

To identify patterns of long-range translocations, which could inform us about chromosome topologies, we took the following coarse-grained approach. We binned each chromosome into four equally sized segments. The first segment, termed ’O’ for the presence of *oriC*, comprised 25% of the chromosome centered around *oriC*. The ’L’ (Left) and the ’R’ (Right) segments each covered 25% of the chromosome on either side of the O segment. The remaining 25%, including the terminus of replication, was termed the ’T’ (Terminus) segment (Figure 2A). Each gene with an ortholog in a partner genome was assigned to one of these four bins.

Analysis of gene functions, for each bin, at a broad level using the COG annotations revealed that the only statistically significant association between gene function and gene positioning was the enrichment of genes involved in translation and ribosome biogenesis in the O (origin) bin (Figure 2B and Figure S7). In line with previous literature (Couturier and Rocha 2006), we observed that the average distance of translation and ribosome biogenesis genes from *oriC* decreases with increase in *R*_f_ (Figure S8). The enrichment of ribosome biogenesis and translation genes in the O segment was particularly apparent in fast-growing bacteria (*R*_f_ > 1) but not in slower growing ones (Figure 2B). In contrast, there was a significant depletion of predicted horizontally acquired genes in the O bin, in both fast- and slow-growing bacteria, and more so in the former (Figure 2C). This is consistent with the increased chance of novel gene integration disrupting essential gene functions in this region of the chromosome. These results are also in agreement with observations made in *E. coli* (Zarei *et al.* 2013) wherein horizontally acquired genes were defined using methods different from what we have used here.

### Long-range interreplichore translocations

The degree of conservation – defined as the proportion of genes with an ortholog in a closely related partner genome – decreased from the O bin toward the T bin for fast-growing organisms, with slow-growing organisms showing little effect (Figure S9).

**Figure 3:**
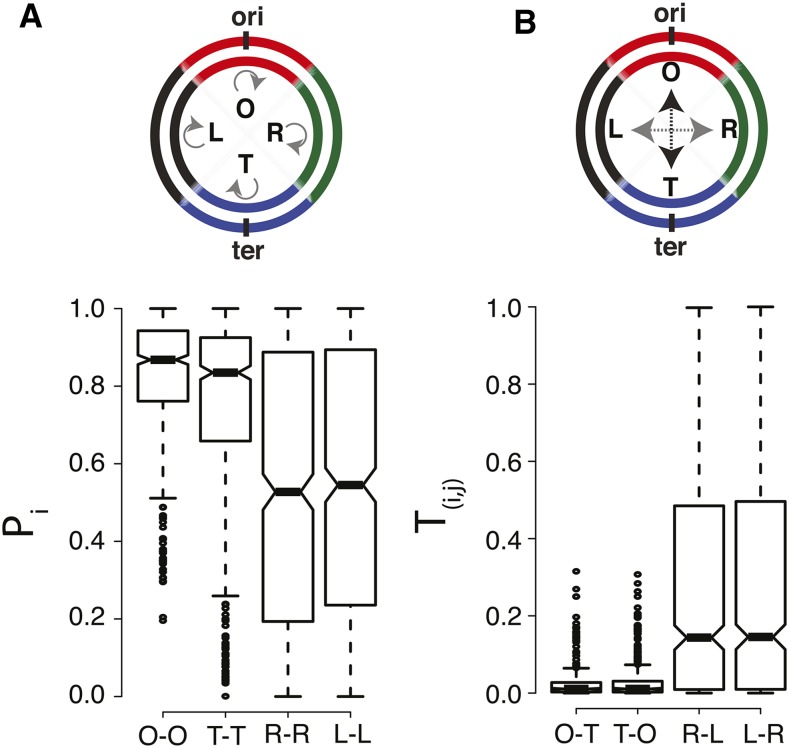
Dynamics of gene organization. (A) Schematic and boxplot showing the conservation of gene position (*P*_i_) in O bin (O−O), T bin (T−T), R bin (R−R), and L bin (L−L). (B) Schematic representing O−T and T−O translocations (black arrows) and L−R and R−L translocations (gray arrows) and boxplot showing the proportion of these translocations (*T*_(i,j)_).

We then asked whether genes present in a given segment of a chromosome would be conserved within the same segment in its closely related partner genome and whether this property would differ across the four segments defined here. We define *P*_i_ – the *gene position conservation* for segment i (where i stands for the four chromosomal segments O, T, R, or L) − as the proportion of genes in that segment, which are conserved in the corresponding segment in its partner genome (Figure 3A). The median *P*_O_ and *P*_T_, for the O and the T bins, across our dataset of 262 genome pairs, was ∼80%, and the distributions of *P*_O_ and *P*_T_ were tight. In contrast, *P*_L_ and *P*_R_ showed a wide distribution, with the median around 50% (Figure 3A).

Next, we asked whether a gene present in a particular bin in one organism translocates to the diametrically opposite bin (or a neighboring bin − Figure S10) in the genome of a closely related organism. We called this *translocation rate* (*T*_i-j_, where i and j indicate the segments between which the translocation rate is measured) (Figure 3B). Consistent with our observation of high *P*_O_ and *P*_T_, we found that *T*_O_*_−_*_T_ and *T*_T_*_−_*_O_ are very low with a median of ∼1% (Figure 3B). This could be a possible consequence of selection acting against translocations that would drastically disrupt gene dosage, and is consistent with the observation of few contacts between *oriC* and *ter*.

We next looked at translocations between the L and the R segments (*T*_L−R_ and *T*_R−L_), which represent interreplichore translocations (Figure 3B). For ∼42% of genome pairs, *T*_L−R/R−L_ <0.1; for the remaining, *T*_L−R/R−L_ ∼0.45 (median). Therefore, in the majority of genome pairs compared here, genes present in one replichore in one genome are often present in the opposite replichore in its partner genome.

Note that in identifying interreplichore translocations, by considering only the L and the R bins, we neglected 25% of each replichore immediately adjacent to *oriC* and *ter*. This was done to ensure that our observations were not skewed by previously reported high inversion rates immediately around *oriC* and *ter* (Eisen *et al.* 2000; Suyama and Bork 2001; Tillier and Collins 2000). Note, however, that the proportion of interreplichore translocations within the O and the T segments correlate well with that between the R and the L segments. Translocations appear to be more frequent in the *ter*-proximal than in the *oriC*-proximal half of the chromosome (Figure S11). The difference in translocation rates between the *oriC*-proximal and the *ter*-proximal halves of a chromosome not only suggests a complementary strategy for studying long-range translocations, but also further corroborates the interplay of chromosome topology and gene organization with the process of replication.

To ensure that the interreplichore translocations we describe are not merely a result of discrepancies in gene annotations, we predicted genes using Glimmer (Delcher *et al.* 1999) for all genomes included in this study. We compared predicted gene start positions with those reported in NCBI and found that the two datasets are in agreement with each other (Figure S12).

In summary, we show that interreplichore translocations between the L and the R bins are probably more common than could be perceived from the literature (Eisen *et al.* 2000; Tillier and Collins 2000). Further, the variation in the distribution of L−R translocation rates is only weakly explained by phylogenetic distance (Figure S13 and Figure S14).

### Interreplichore translocations as a possible readout of chromosome topology

Rates of recombination have been used as a proxy for measuring contact frequencies between distant regions of a chromosome. Together with the higher resolution analysis of contact frequencies by chromosome conformation capture methods and microscopy, these have so far underscored two broad chromosome topologies. First, in *E. coli* it has been shown that *oriC* is localized to the midcell and the left and the right arms of the chromosome stretch toward opposite poles with few interreplichore contacts (Nielsen *et al.* 2006; Wang *et al.* 2006). In line with this, we observed low interreplichore translocations (*T*_L−R/R−L_ ∼0.03) between *E. coli* and *Salmonella* (two closely related bacteria).

In contrast, in *C. crescentus* (Le *et al.* 2013; Umbarger *et al.* 2011) and *M. smegmatis* (Santi and McKinney 2015), *oriC* and *ter* are present at the two poles of the cell and the left and the right arms of the chromosome are in close contact with each other. Both these genomes showed high interreplichore translocations (*T*_L−R/R−L_ ∼0.50 and ∼0.70, respectively), consistent with their chromosome topology.

**Figure 4:**
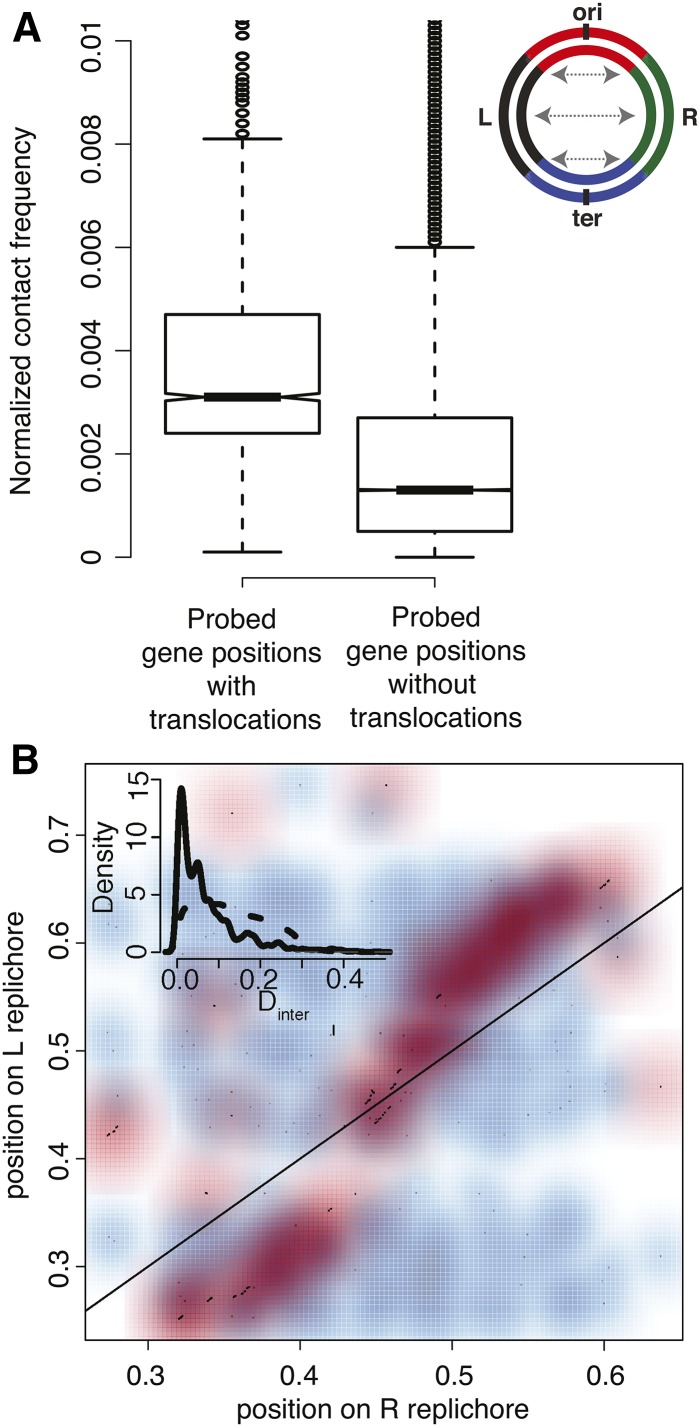
Interreplichore translocations and chromosomal contact points. (A) Boxplot representing the normalized interreplichore contact frequencies as derived from Le *et al.* (2013) for *C. crescentus* NA1000 (NC_011916); for all the probed gene positions with or without interreplichore translocations (*P*-value < 10^−10^, Wilcoxon test). (B) Scatterplot representing 386 interreplichore (R → L) translocations between *C. crescentus* NA1000 (NC_011916) and *C. segnis* (NC_014100) in red and randomized dataset (as described in *Materials and Methods*) in blue. Inner panel shows the distribution of *D*_inter_ = | *d*_Ri/Lj_ – *d*_Li/Rj_ | (solid line) for interreplichore translocations between *C. crescentus* NA1000 (NC_011916) and *C. segnis* (NC_014100). Dashed line indicates distribution of *D*_inter_ for randomized data (as described in *Materials and Methods*).

Next, we analyzed the chromosome contact map from a previously published Hi-C experiment for *C. crescentus* (Le *et al.* 2013). Pairs of loci translocated between the two replichores displayed significantly higher contact frequencies in Hi-C experiments when compared to pairs of interreplichore loci not showing translocations (Figure 4A and Figure S15). These indicate that high rates of translocation are localized to spatially proximal regions of the chromosome.

**Figure 5:**
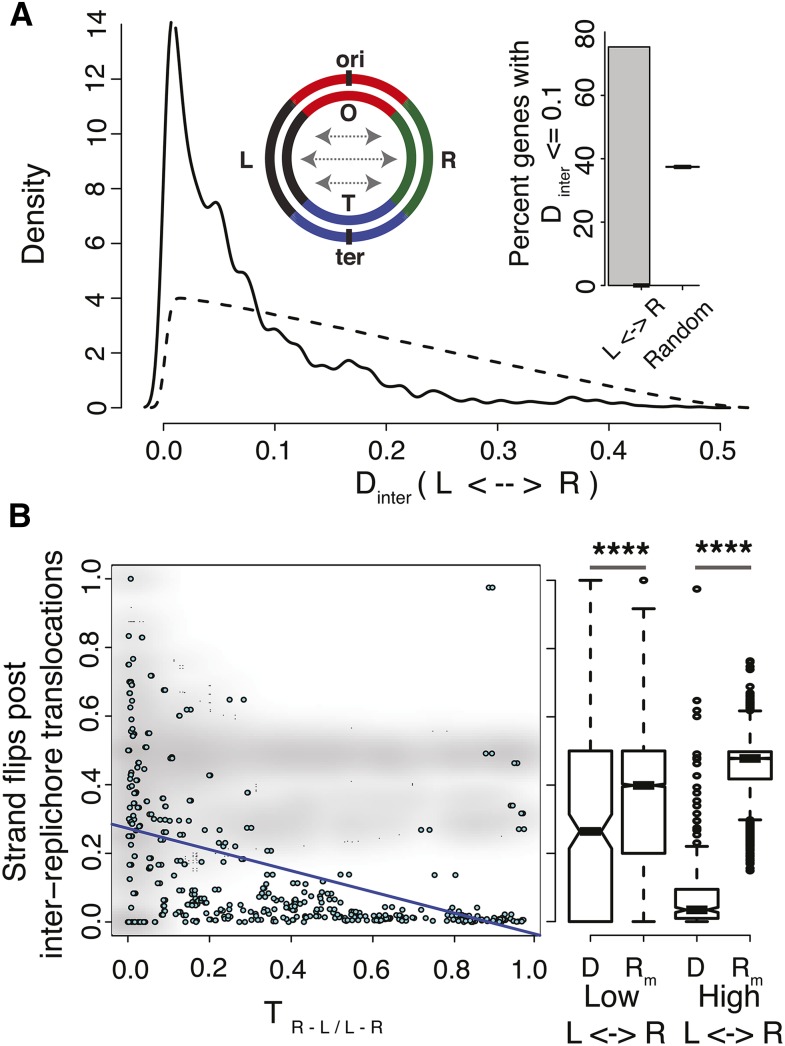
Selective value of chromosome topology. (A) Plot representing the distribution of *D*_inter_ = |*d*_R/L_ – *d*_L/R_| for all bacteria showing high interreplichore translocations (*T*_L−R/R−L_ > 0.1). Dashed line indicates the distribution of *D*_inter_ for randomized datasets (as described in *Materials and Methods*), similar to the inner panel of Figure 4B. Inner panel shows the percentage of gene pairs with *D*_inter_ < 0.1 for all bacteria showing high interreplichore translocations, alongside random expectation (determined from randomized datasets as described in *Materials and Methods*). (B) Plot representing the proportion of leading-lagging strand flips post interreplichore translocations as a function of the proportion of interreplichore translocations (ρ_Spearman_ = 0.17, *P*-value < 10^−10^) in cyan. A randomized dataset (as described in *Materials and Methods*) is shown in gray. Adjacent bloxplot represents the distribution of leading-lagging strand flips in bacteria with high and low interreplichore translocations alongside random expectation (*R*_m_) for each dataset (D) (low *T*_L−R/R−L_, *P*-value = 2.9 × 10^−6^, Wilcoxon test and high *T*_L−R/R−L_, *P*-value < 10^−10^,Wilcoxon test). Asterisks indicate *P*-value < 10^−3^.

Many interreplichore translocations in *C. crescentus* (Figure 4B) and in *M. smegmatis* (Figure S16A) are symmetric, *i.e.*, a gene at a certain distance *d*_R_ from *oriC* in one replichore translocates to a position *d*_L_ in the other replichore such that *D*_inter_ *= |d*_R_ *− d*_L_*|* is close to zero. This is again consistent with the topology of these chromosomes, and also with the idea that a pair of concurrent replication forks, which are typically equidistant from *oriC*, are hotspots for recombination. Such symmetric translocations are true, not only for *C. crescentus* and *M. smegmatis*, but are a general feature of genomes with high *T*_L−R/R−_*_L_* values (Figure 5A), but less so for genome pairs with low *T*_L−R/R−L_ (*T*_L−R/R−L_ < 0.1) (Figure S16B). These are in line with the high ρ_GOC_ values across most genome pairs tested here, indicating that interreplichore translocations rarely disrupt the relative distance of genes from *oriC*, and thereby maintain their dosage.

We also found that interreplichore translocations do not impact strand bias in gene densities. We noticed that the translocated partners of leading strand genes tend to be encoded on the leading strand of the opposite replichore. This is more prominent for organisms showing high *T*_L−R/R−L_ as compared to low *T*_L−R/R−L_ (Figure 5B). This difference in strand maintenance between genomes with high and low *T*_L−R/R−L_ values might point to mechanistic differences between the two groups of organisms in the way these translocations occur.

Finally, we observed that interreplichore translocations rarely break operons. However, the contiguity of genes belonging to the same functional category is often broken by these translocations (Figure S17). In order to explicitly test the impact of these interreplichore translocations on gene expression states, we obtained publicly available microarray data for *Shewanella oneidensis MR1* (high *T*_L−R/R−L_; *T*_L−R/R−L_ ∼0.56) across many conditions from the M3D database (http://m3d.mssm.edu). Analysis of these microarray data showed that genes present on different replichores show a higher correlation in expression (ρ_Spearman_ > 0.5) when they are equidistant from *oriC*. In contrast, gene pairs showing little or negative correlations between their gene expression profiles tend to show greater differences in their distance from *oriC*. Although the effect is very weak, the trend is statistically significant in terms of the difference between medians (*P*-value < 10^−10^, Wilcoxon test; Figure S18). This suggests that interreplichore translocations between genes equidistant from *oriC* may not disrupt their gene expression states.

Thus, interreplichore translocations − potentially encouraged by the spatial proximity of the two replichores − neither disrupt gene dosage gradients down the *oriC*−*ter* axis nor do they impact strand biases in gene densities (Figure S22); they may have only a small effect on gene expression states.

## Discussion

Genome architecture broadly refers to gene organization and chromosome topology. While the wealth of genome sequence data provides valuable insights into gene organization, interrogation of chromosome topologies at high resolution requires additional experimental techniques involving chromosome conformation capture (Dekker *et al.* 2002). These techniques were deployed on a genome-wide scale initially for eukaryotes and are now being used to study prokaryotic genome structures (Cagliero *et al.* 2013; Le *et al.* 2013; Marbouty *et al.* 2014, 2015; Umbarger *et al.* 2011). These studies have been limited so far to studying the three-dimensional chromosome architectures of a few model organisms. This currently precludes further analysis of associations between gene order and chromosome topologies.

In this study, we have used a comparative genomic framework to indicate that large-scale translocations over short phylogenetic distances could be a proxy for chromosome topologies. This is consistent with the observation, in eukaryotes, that various cancer-causing translocations in the human genome involve loci with high contact frequencies in normal tissue (Engreitz *et al.* 2012). We notice that genomes with high interreplichore translocations may also experience high rates of intrareplichore recombination. Whether this implicates symmetric interreplichore chromosomal contacts in the maintenance of gene order is hard to address with confidence (Figure S19A). It is however not clear as to what extent chromosome topology reflects phylogeny, as we observe a wide variation in the rates of interreplichore translocations within phylogenetic classes (Figure S20). Additionally, we do not observe any association between growth rates and interreplichore translocation rates. And in light of our suggestion that high rates of interreplichore translocations may be predictive of a longitudinal chromosome topology, the growth rate of an organism may not determine its global chromosome topology (Figure S21).

At this resolution, our analysis might predict whether the two replichores of a bacterial chromosome are spatially colocalized, but does not say anything about finer aspects of its structuring, such as macro- and microdomains. For example, there are only a few long-range translocations in the genome of *E. coli*; many of these appear to be located around *ter*, which is closely organized by the binding of the protein MatP (Dupaigne *et al.* 2012) and where many targets of the nucleoid-associated protein H-NS are localized. Even the *C. crescentus* chromosome has local domain structures in the 20−400 kb size range (Le *et al.* 2013). However, these are not predicted by our translocation analysis.

Another caveat of our study is that the dynamics of chromosome topology may not be predictable. For example, recent studies in *B. subtilis* have shown that its chromosome oscillates between the longitudinal and the transverse topologies (X. Wang *et al.* 2014b). During replication *B. subtilis* is shown to have the *E. coli*-like transverse topology and the *C. crescentus*-like longitudinal topology for the rest of the cell cycle. However, *B. subtilis* shows interreplichore translocation frequency (∼0.02) similar to that of *E. coli*. This is consistent with the notion that concurrent replication forks are hotspots for recombination. Therefore, interreplichore translocations might occur during replication, and may be facilitated by close spatial contacts between the two replichores during replication. These observations hint toward the existence of a strong interplay between replication and chromosome topology. Hence, we suggest that the association between chromosome topologies and growth phenotypes, and its mechanistic and evolutionary underpinnings, should be explored in greater detail.

Recent studies have shown that genome architecture and gene expression are linked in the following ways: (a) through differential positioning of genes with respect to *oriC*, popularly referred to as the gene dosage effect (Block *et al.* 2012; Bryant *et al.* 2014; Narula *et al.* 2015; Schmid and Roth 1987); (b) through spatial clustering of nonconsecutive genes, which might enforce transcriptional coherence (Cabrera and Jin 2003; Junier *et al.* 2012; Qian *et al.* 2012; Wang *et al.* 2011); (c) through preferential binding of bacterial nucleoid-associated proteins like H-NS, which show position and gene feature-specific binding to the chromosome (Tupper *et al.* 1994; Weng and Xiao 2014); and (d) through the effect of proposed gradients of DNA superhelicity on the chromosome on gene expression (Lal *et al.* 2016; Sobetzko *et al.* 2012). However, despite our attempts to interrogate this using a compendium of microarray data for one organism, the impact of interreplichore translocations on gene expression states remains open. Along this direction, it remains to be seen whether genomes showing high interreplichore translocations would also display symmetry in nucleoid organization between the two replichores; this could also serve to minimize the impact of such translocations on gene expression. Detailed analyses of the impact of replication on gene expression and chromosome topologies are also essential, in the light of evidence that *oriC* might itself be a selfish element (Hawkins *et al.* 2013), and the ability of bacteria to undergo dispersed replication events independent of *oriC* (Bernander *et al.* 1991; Dimude *et al.* 2015; Gowrishankar 2015; Koppes 1992; McGeoch and Bell 2008).

## Supplementary Material

Supplemental Material
