# Supplementary material for: Comparative Genomics of Interreplichore Translocations in Bacteria: A Measure of Chromosome Topology?
Source: G3 (Bethesda). 2016 Mar 30;6(6):1597–606. doi: 10.1534/g3.116.028274 (PMC4889656; doi:10.1534/g3.116.028274)
Supplement: Supplemental Material [file supp_6_6_1597__index.html]

Supplemental Material 

# Comparative Genomics of Interreplichore Translocations in Bacteria: A Measure of Chromosome Topology?

Supplemental Material for Khedkar and Seshasayee, 2016

Supplemental Material

**Files in this Data Supplement:**

- Figure S1 - Plot representing the predicted values of R-factor (Rf) using two different datasets. (.pdf, 243 KB)
- Figure S2 - A) Boxplot representing the distribution of 16S rRNA sequence identity for all bacterial pairs, bacterial pairs belonging to the same genus and those belonging to the same species. (.pdf, 385 KB)
- Figure S3 - A) Density plot representing the proportion of TL-R / R-L in the 262 pairs of bacteria used in this study (solid line) and the proportion of TL-R / R-L in ~100 pairs of bacteria belonging to the same species (dashed line); B) Same distributions as in (A) represented using boxplot. (.pdf, 197 KB)
- Figure S4 - 16S rRNA phylogenetic tree deduced from 16S rRNA sequence of the 232 bacterial species used in this study. (.pdf, 8 MB)
- Figure S5 - A) Plot showing the dependence of the fraction of genes conserved between genome pairs on the phylogenetic distance between the genomes compared; this plot was used to fit a LOESS curve between the two axes, and the residual of fit computed. (.pdf, 518 KB)
- Figure S6 - A) Plot showing the dependence of Gene Order Conservation (GOC) on phylogenetic distance in closely-related bacteria; this plot was used to fit a LOESS curve between the two axes, and the residual of fit computed. (.pdf, 611 KB)
- Figure S7 - Boxplot representing the negative log (base 10) transformed Bonferroni corrected p-values of Fisher exact test (described in methods) performed to determine the enrichment of 24 Cluster of Orthologous Genes (COG) categories in the O bin. (.pdf, 497 KB)
- Figure S8 - Plot representing the average distance of translation and ribosome biogenesis genes from *oriC* as a function of Rf. (.pdf, 516 KB)
- Figure S9 - A) Boxplot representing the proportion of genes conserved in different chromosomal bins: Origin bin - O, Right bin- R, Terminus bin - T and Left bin - L for all 262 pairs of bacteria. (.pdf, 288 KB)
- Figure S10 - Boxplot representing the proportion of genes translocating to the same bin and across different chromosomal bins (O, T, R and L). (.pdf, 714 KB)
- Figure S11 - A) Plot representing the proportion of inter-replichore translocations between RO and LO (as per schematic) versus translocations within the O bin, but across replichores (OR and OL) (ρSpearman = 0.8, *P*-value < 10-10). (.pdf, 1 MB)
- Figure S12 - Plot representing the difference in the number of base pairs between the gene start sites predicted using Glimmer (Delcher *et al*. 1999) and the gene start sites as reported by NCBI. (.pdf, 171 KB)
- Figure S13 - (A, left) Plot representing the dependence of TO-T on phylogenetic distance; (A, middle) Plot representing the absence of dependence of residual TO - T on phylogenetic distance after correcting for phylogenetic distance. (.pdf, 1 MB)
- Figure S14 - A) Plot representing the relationship between TR-L and the residual of TR-L after correcting for its dependence on phylogenetic distance. (.pdf, 540 KB)
- Figure S15 - Boxplot representing the normalized inter-replichore contact frequencies between R and L bins as derived from (Le *et al*. 2013) for all probed gene positions with and without inter-replichore translocations in *Caulobacter crescentus* NA1000 (NC\_011916) (*P*-value < 10-10, Wilcoxon test). (.pdf, 212 KB)
- Figure S16 - A) Scatterplot representing 727 inter-replichore translocations in red between *Mycobacterium sp*. (NC\_014814) and *Mycobacterium smegmatis* (NC\_008596). (.pdf, 667 KB)
- Figure S17 - Boxplot representing inter-replichore translocations leading to three kinds of breakpoints 1) functional breaks - breaks in contiguous genes which are functionally similar; 2) Operon breaks - breaks disrupting contiguity of genes belonging to an operon and 3) Whole Operon transfers - breaks that do not disrupt the contiguity of genes belonging to an operon. (.pdf, 160 KB)
- Figure S18 - Boxplot representing the distribution of D*inter* (difference in distance of genes present on two different replichores from *oriC*) in *Shewanella oneidensis* MR1 for different values of correlation in gene expression levels. (.pdf, 210 KB)
- Figure S19 - To make the selection of orthologs more stringent, we selected a subset of orthologs that showed >=80% sequence identity. We repeated the key analyses described in results using this subset alone. (.pdf, 608 KB)
- Figure S20 - A) Boxplot representing gene movement as residual (Dintra). (.pdf, 783 KB)
- Figure S21 - Boxplot representing the distribution of TL-R/R-L in Alpha and Gamma proteobacteria. TL-R/R-L values for *Caulobacter crescentus* are marked in red under Alphaproteobacteria. T*L-R/R-L* values for *E. coli* are marked in red under Gamma-proteobacteria. (.pdf, 174 KB)
- Figure S22 - Boxplot representing the distribution of Rf for bacteria with low and high interreplichore translocations (*P*-value = 0.03, Wilcoxon test). (.pdf, 152 KB)
- File S1 - COG categories (.pdf, 49 KB)
- File S2 - References for supplemental materials. (.pdf, 62 KB)
