# Supplementary material for: Comparative Genomics of Interreplichore Translocations in Bacteria: A Measure of Chromosome Topology?
Source: G3 (Bethesda). 2016 Mar 30;6(6):1597–606. doi: 10.1534/g3.116.028274 (PMC4889656; doi:10.1534/g3.116.028274)
Supplement: Supplemental Material [file supp_g3.116.028274_FigureS1.pdf]

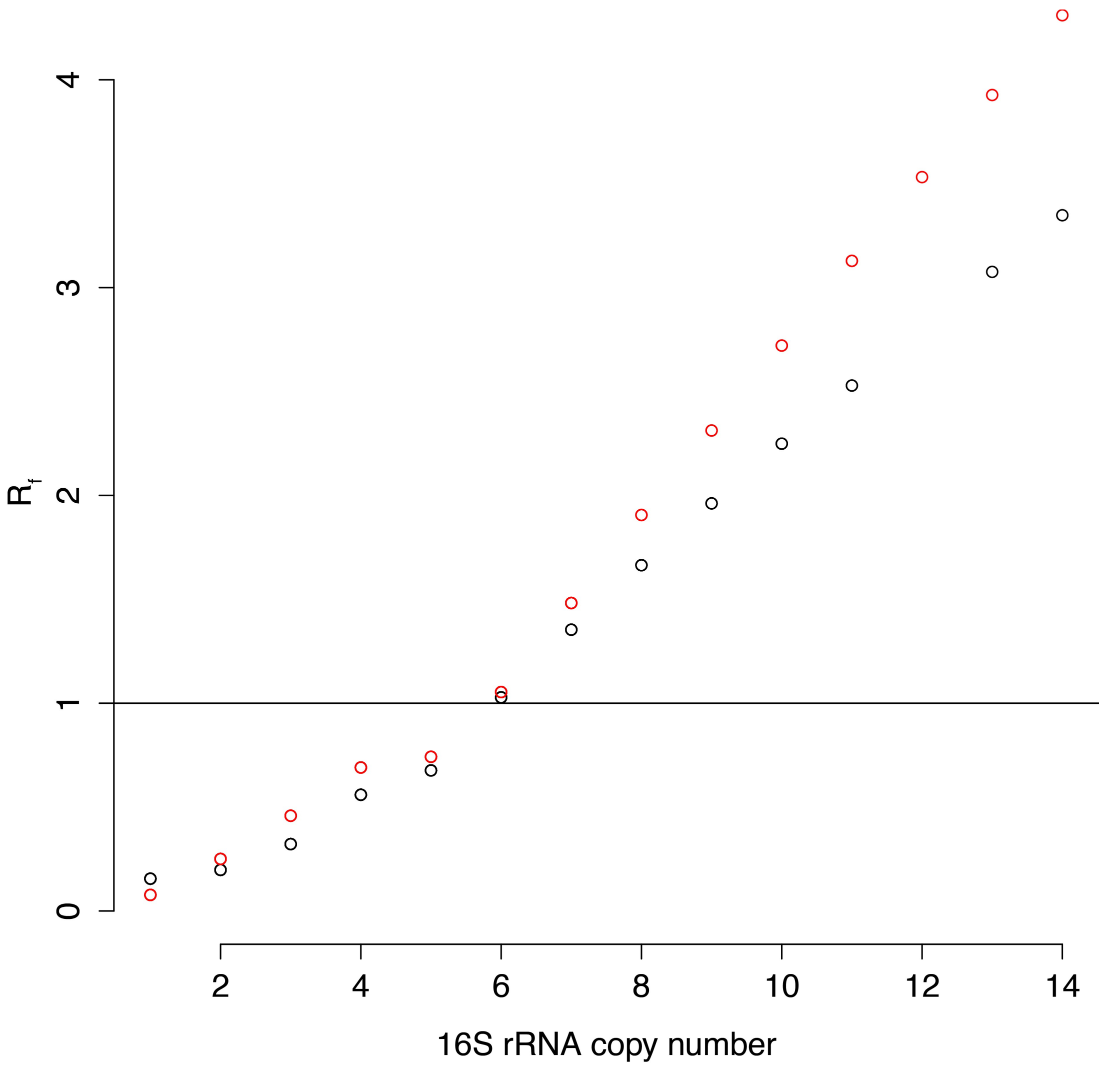

Figure S1 Plot representing the predicted values of R-factor (R_f) using two different datasets. The dataset used in our study, published by (Freilich *et al.* 2009) is shown in red. A newer dataset by (Vieira-Silva and Rocha 2010) is in black.
