# Supplementary figures and images for: Comparative Genomics of Interreplichore Translocations in Bacteria: A Measure of Chromosome Topology?
Source: G3 (Bethesda). 2016 Mar 30;6(6):1597–606. doi: 10.1534/g3.116.028274 (PMC4889656; doi:10.1534/g3.116.028274)

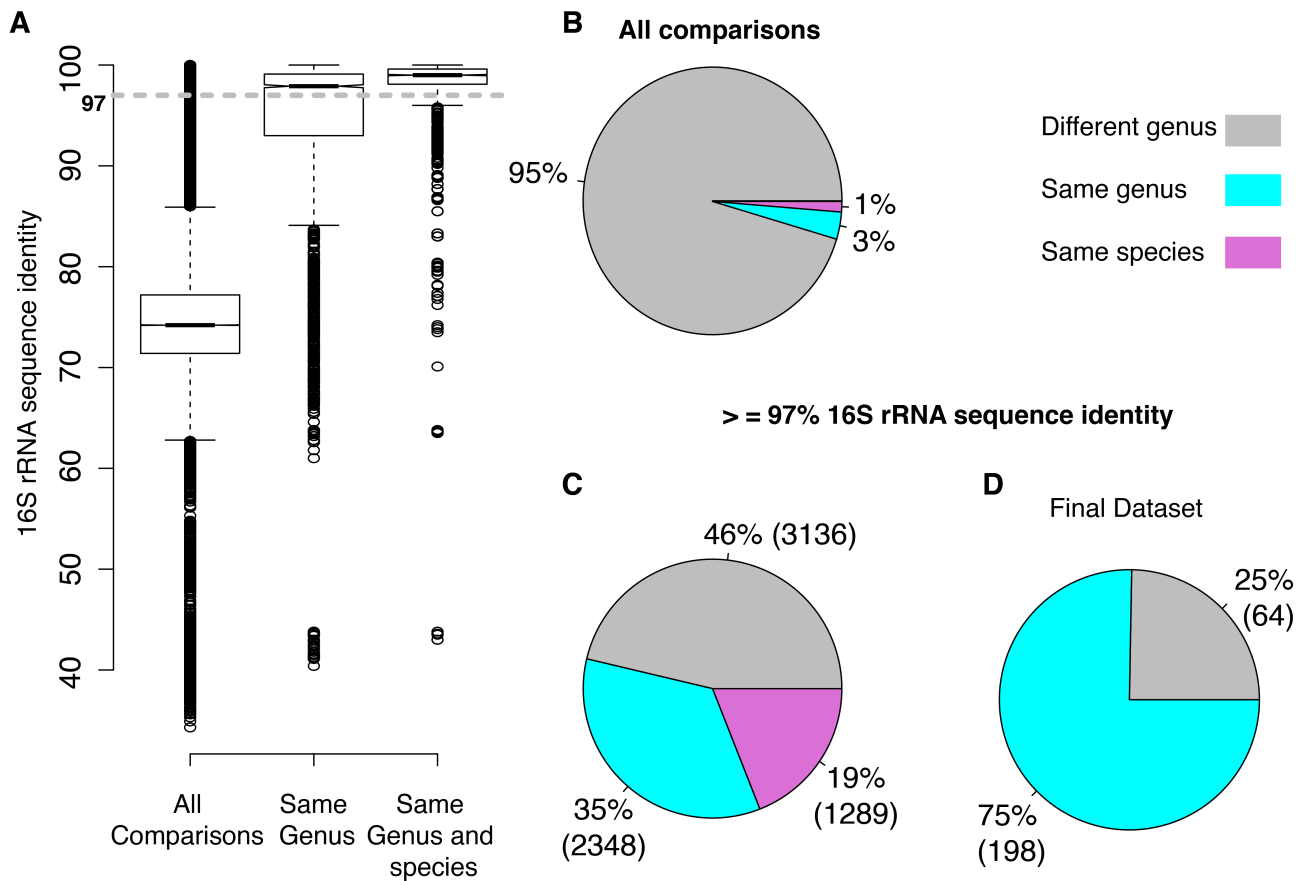

Supplement: Supplemental Material [file supp_g3.116.028274_FigureS2.pdf]
