# Supplementary material for: Comparative Genomics of Interreplichore Translocations in Bacteria: A Measure of Chromosome Topology?
Source: G3 (Bethesda). 2016 Mar 30;6(6):1597–606. doi: 10.1534/g3.116.028274 (PMC4889656; doi:10.1534/g3.116.028274)
Supplement: Supplemental Material [file supp_g3.116.028274_FigureS3.pdf]

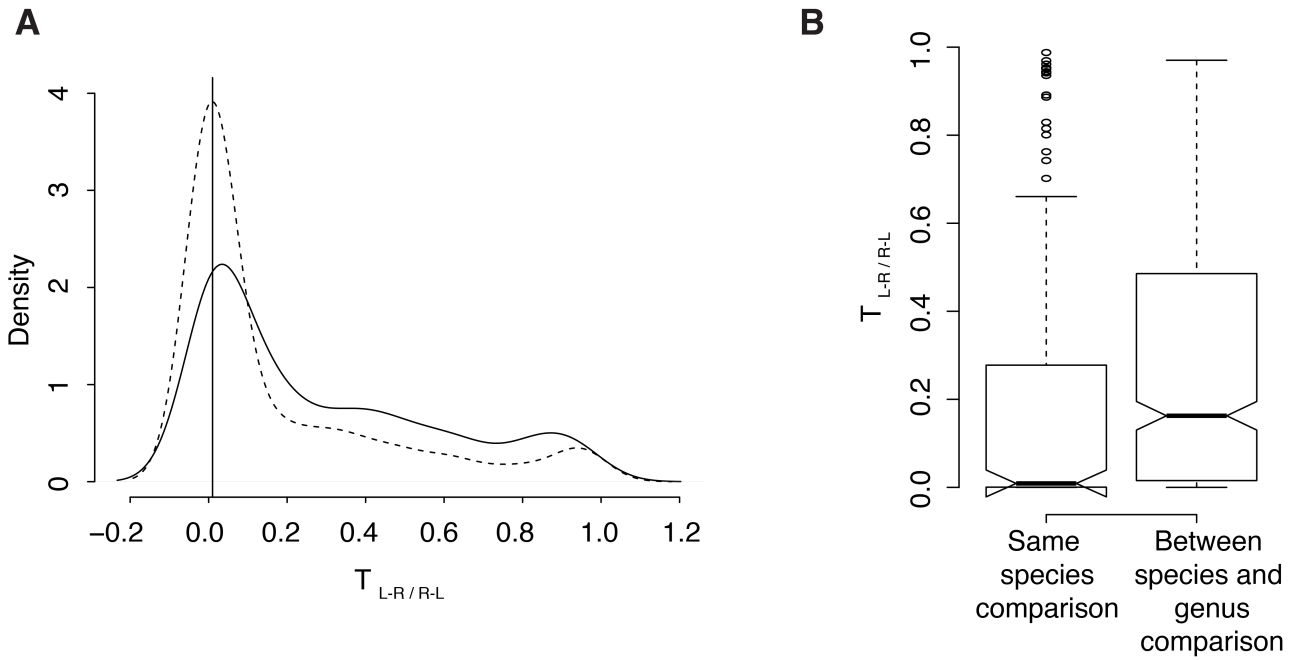

Figure S3 A) Density plot representing the proportion of $T_{L\text{-}R\,/\,R\text{-}L}$ in the 262 pairs of bacteria used in this study (solid line) and the proportion of $T_{L\text{-}R\,/\,R\text{-}L}$ in ~100 pairs of bacteria belonging to the same species (dashed line); B) Same distributions as in (A) represented using boxplot.
