# Supplementary material for: Comparative Genomics of Interreplichore Translocations in Bacteria: A Measure of Chromosome Topology?
Source: G3 (Bethesda). 2016 Mar 30;6(6):1597–606. doi: 10.1534/g3.116.028274 (PMC4889656; doi:10.1534/g3.116.028274)
Supplement: Supplemental Material [file supp_g3.116.028274_FigureS4.pdf]

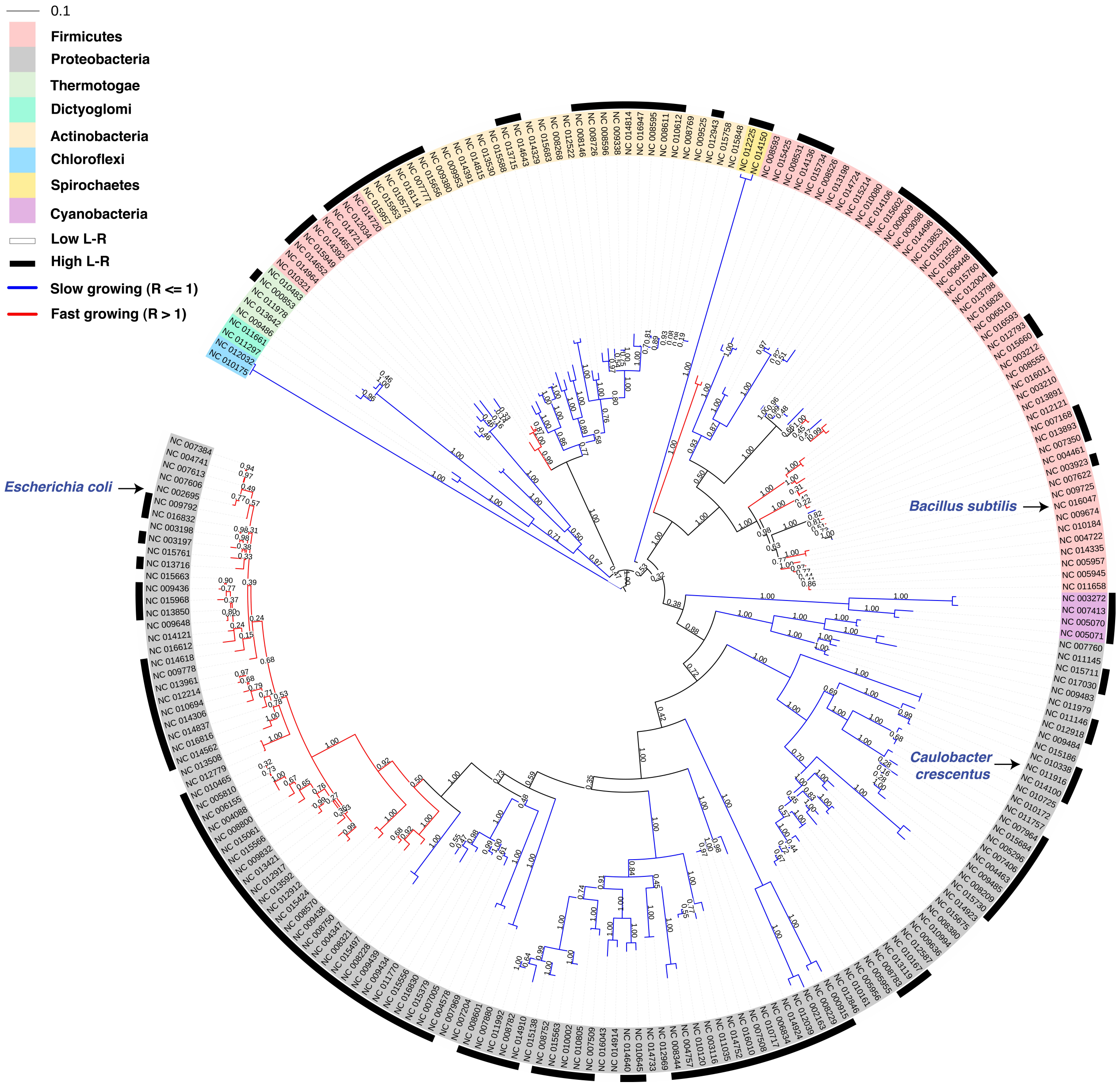

Figure S4 16S rRNA phylogenetic tree deduced from 16S rRNA sequence of the 232 bacterial species used in this study. 16S rRNA sequence of the rRNA copy closest to the origin of replication was obtained from the genome sequences of the 232 bacteria. A multiple sequence alignment was generated using MUSCLE (Edgar 2004) alignment tool, implemented in MEGA version 6. A Maximum Likelihood (ML) tree was generated using all sites in the 16S rRNA alignment. Tamura and Nei (Tamura and Nei 1993) nucleotide substitution model was used to infer the tree. The phylogenetic tree was visualized using iTOL and growth rate information and inter-replichore translocation frequency were overlaid on the obtained phylogenetic tree. Different bacterial phyla are color coded as represented in the legend. The numbers on the tree above branches indicate bootstrap values (100 replications).
