# Supplementary material for: Comparative Genomics of Interreplichore Translocations in Bacteria: A Measure of Chromosome Topology?
Source: G3 (Bethesda). 2016 Mar 30;6(6):1597–606. doi: 10.1534/g3.116.028274 (PMC4889656; doi:10.1534/g3.116.028274)
Supplement: Supplemental Material [file supp_g3.116.028274_FigureS5.pdf]

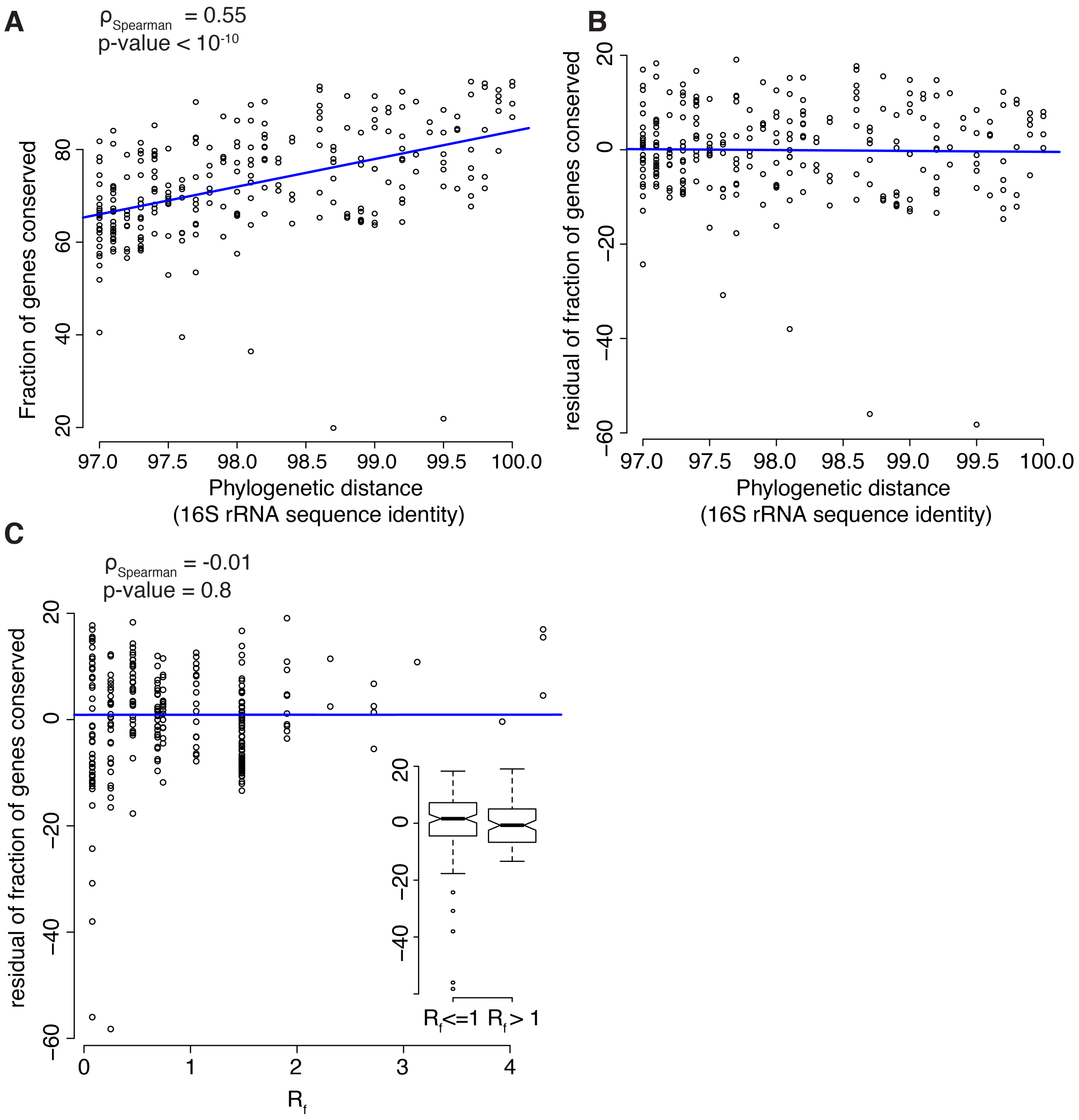

Figure S5 A) Plot showing the dependence of the fraction of genes conserved between genome pairs on the phylogenetic distance between the genomes compared; this plot was used to fit a LOESS curve between the two axes, and the residual of fit computed. B) Plot representing the absence of a correlation between the residual of the fraction of genes conserved on phylogenetic distance; C) Plot showing the residual of the fraction of genes conserved as a function of R-factor (R_f). Inner panel shows no significant difference between residual of fraction of conserved genes between slow ($R_f <= 1$) and fast ($R_f > 1$) growing bacteria.
