# Supplementary material for: Comparative Genomics of Interreplichore Translocations in Bacteria: A Measure of Chromosome Topology?
Source: G3 (Bethesda). 2016 Mar 30;6(6):1597–606. doi: 10.1534/g3.116.028274 (PMC4889656; doi:10.1534/g3.116.028274)
Supplement: Supplemental Material [file supp_g3.116.028274_FigureS6.pdf]

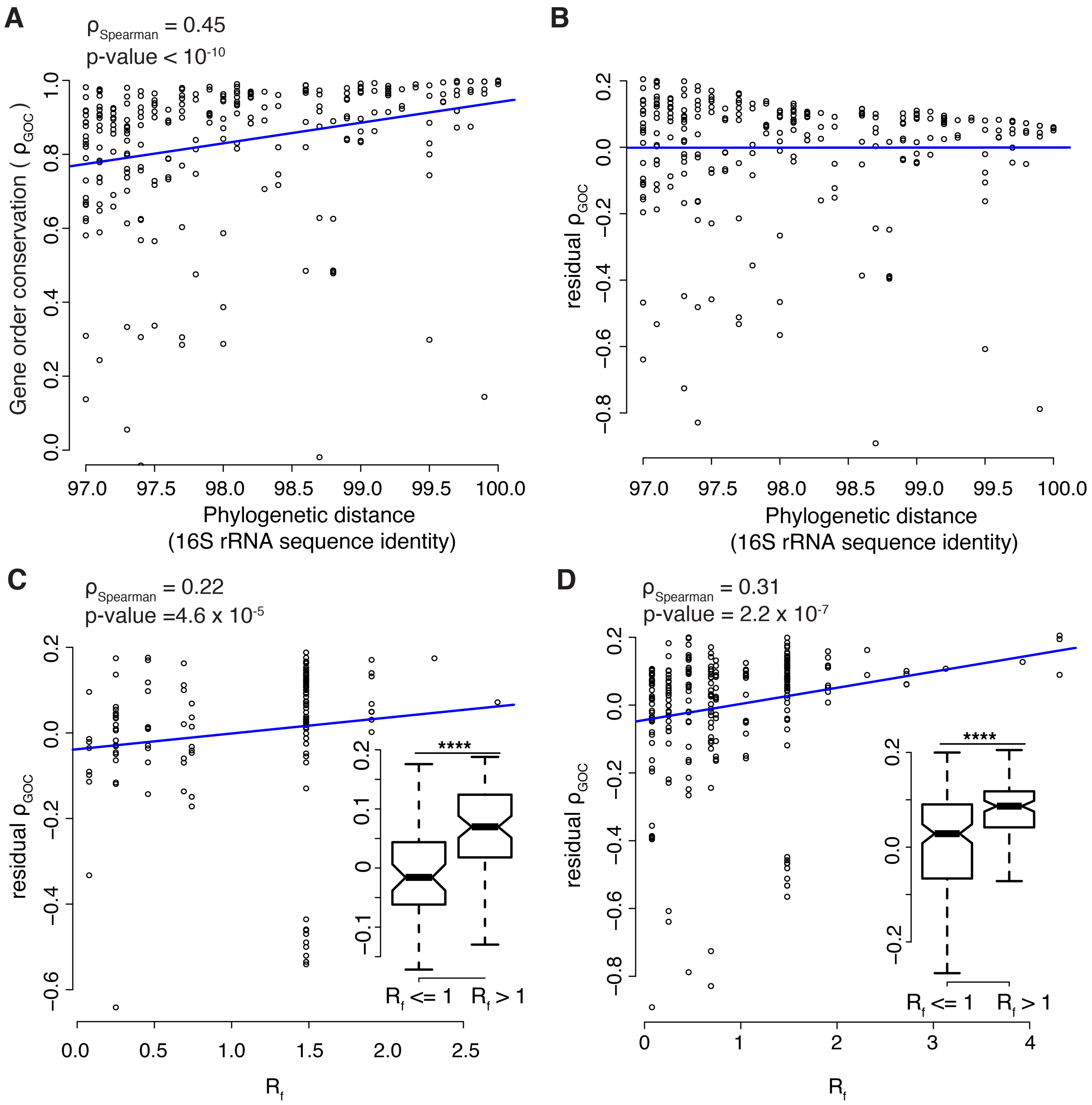

Figure S6 A) Plot showing the dependence of Gene Order Conservation (GOC) on phylogenetic distance in closely-related bacteria; this plot was used to fit a LOESS curve between the two axes, and the residual of fit computed. B) Plot representing the absence of a correlation between residual ρ_{GOC} and phylogenetic distance; C) Plot showing the correlation between residual ρ_{GOC} and R_f for bacteria belonging to the phylum Proteobacteria. Inner panel shows a statistically significant difference between residual ρ_{GOC} for slow ($R_f <= 1$) and fast ($R_f >1$) growing Proteobacteria (*P*-value = 1×10^{-4}, Wilcoxon test); D) Plot representing the correlation between residual ρ_{GOC} and R_f for bacteria belonging to phyla other than Proteobacteria. Inner panel showing a statistically significant difference between residual ρ_{GOC} for slow ($R_f <= 1$) and fast ($R_f > 1$) growing non-Proteobacteria (*P*-value = 3.3×10^{-6}, Wilcoxon test). Asterisks indicate p-value < 10^{-3}.
