# Supplementary material for: Comparative Genomics of Interreplichore Translocations in Bacteria: A Measure of Chromosome Topology?
Source: G3 (Bethesda). 2016 Mar 30;6(6):1597–606. doi: 10.1534/g3.116.028274 (PMC4889656; doi:10.1534/g3.116.028274)
Supplement: Supplemental Material [file supp_g3.116.028274_FigureS7.pdf]

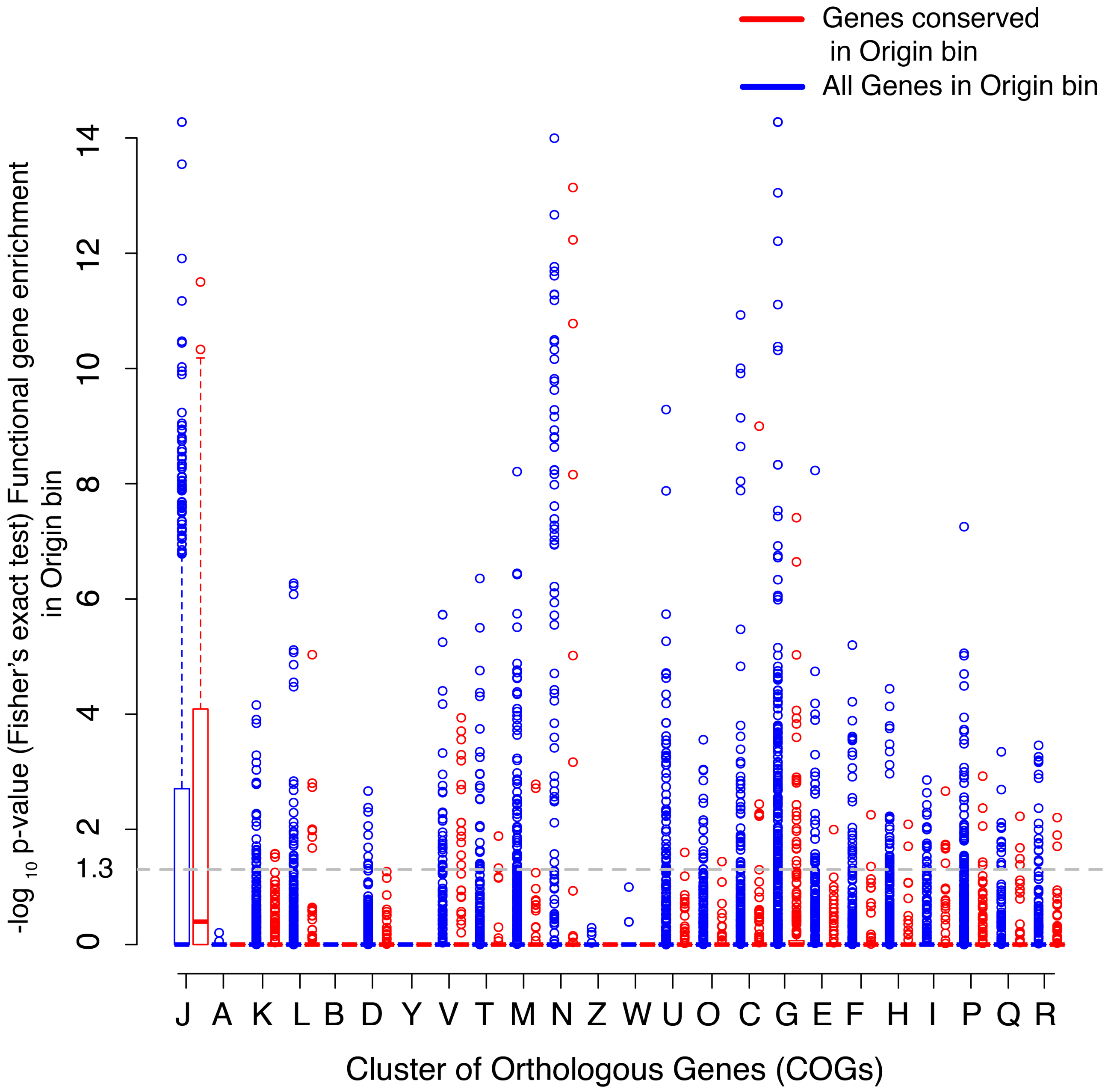

Figure S7 Boxplot representing the negative log (base 10) transformed Bonferroni corrected p-values of Fisher exact test (described in methods) performed to determine the enrichment of 24 Cluster of Orthologous Genes (COG) categories in the O bin. The values in blue are for all the genes present in the O bin and values in red are for genes conserved, in the O bin, between two closely-related bacteria. The dashed grey line at y = 1.3 corresponds to a p-value of 0.05.
