# Supplementary material for: Comparative Genomics of Interreplichore Translocations in Bacteria: A Measure of Chromosome Topology?
Source: G3 (Bethesda). 2016 Mar 30;6(6):1597–606. doi: 10.1534/g3.116.028274 (PMC4889656; doi:10.1534/g3.116.028274)
Supplement: Supplemental Material [file supp_g3.116.028274_FigureS8.pdf]

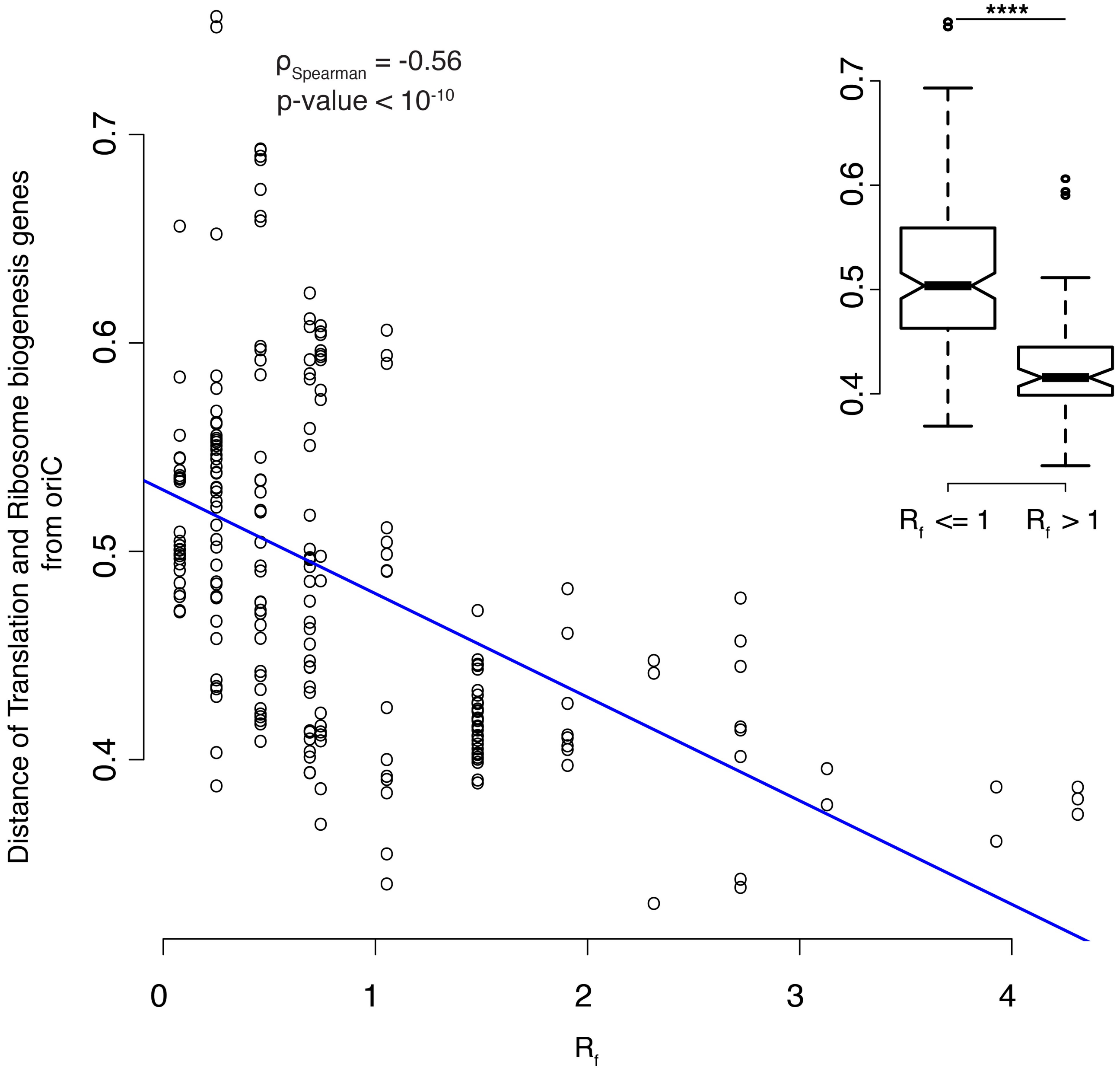

Figure S8 Plot representing the average distance of translation and ribosome biogenesis genes from *oriC* as a function of R_f. Inner panel shows that the average distance of translation and ribosome biogenesis genes from *oriC* is significantly different between slow (R_f <= 1) and fast (R_f > 1) growing bacteria (*P*-value < 10-10, Wilcoxon test). Asterisks indicate pvalue < 10^{-3}.
