# Supplementary material for: Comparative Genomics of Interreplichore Translocations in Bacteria: A Measure of Chromosome Topology?
Source: G3 (Bethesda). 2016 Mar 30;6(6):1597–606. doi: 10.1534/g3.116.028274 (PMC4889656; doi:10.1534/g3.116.028274)
Supplement: Supplemental Material [file supp_g3.116.028274_FigureS9.pdf]

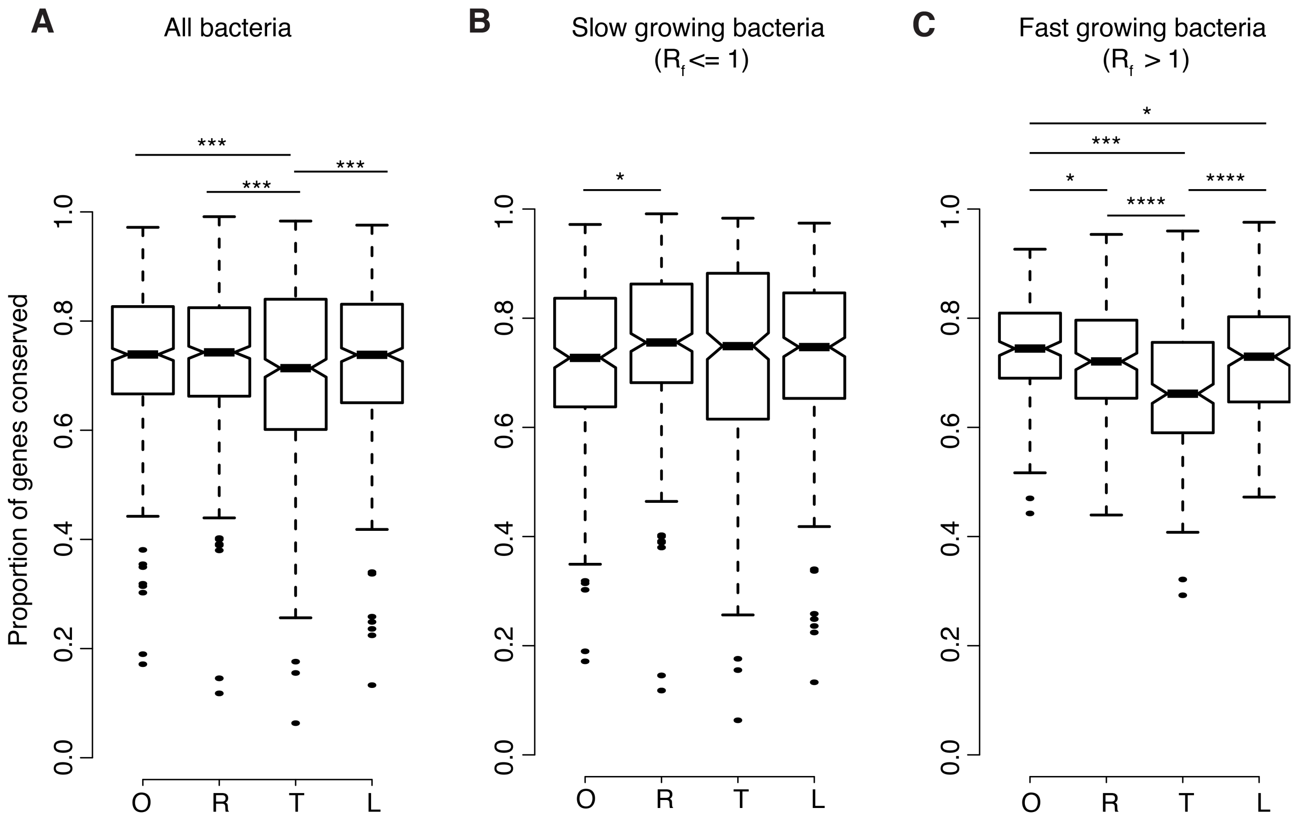

Figure S9 A) Boxplot representing the proportion of genes conserved in different chromosomal bins: Origin bin – O, Right bin- R, Terminus bin - T and Left bin - L for all 262 pairs of bacteria. Statistically significant difference is observed between the proportion of orthologs conserved between O and T bins (*P*-value = 1 x 10^{-3}, Wilcoxon test), R and T bins (*P*-value = 1 x 10^{-3}, Wilcoxon test), and L and T bins (*P*-value = 3 x 10^{-3}, Wilcoxon test); B) Boxplot representing the proportion of orthologs conserved in different chromosomal bins (O, R, T and L) for all pairs of slow (R_f <= 1) growing bacteria. Weakly significant difference is observed between the proportion of orthologs conserved between O and R bins (*P*-value = 0.04, Wilcoxon test); C) Boxplot representing the proportion of orthologs conserved in different chromosomal bins (O, R, T and L) for all pairs of fast (R_f > 1) growing bacteria. Statistically significant difference is observed between the proportion of orthologs conserved between O and T bins (*P*-value < 10^{-10}, Wilcoxon test), O and R bins (*P*-value = 2 x 10^{-3}, Wilcoxon test), O and L bins (*P*-value = 0.02, Wilcoxon test), R and T bins (*P*-value = 6.9 x 10^{-6}, Wilcoxon test), and L and T bins (*P*-value = 3.3 x 10^{-7}, Wilcoxon test). Asterisks indicate p-values.
