# Supplementary material for: Comparative Genomics of Interreplichore Translocations in Bacteria: A Measure of Chromosome Topology?
Source: G3 (Bethesda). 2016 Mar 30;6(6):1597–606. doi: 10.1534/g3.116.028274 (PMC4889656; doi:10.1534/g3.116.028274)
Supplement: Supplemental Material [file supp_g3.116.028274_FigureS10.pdf]

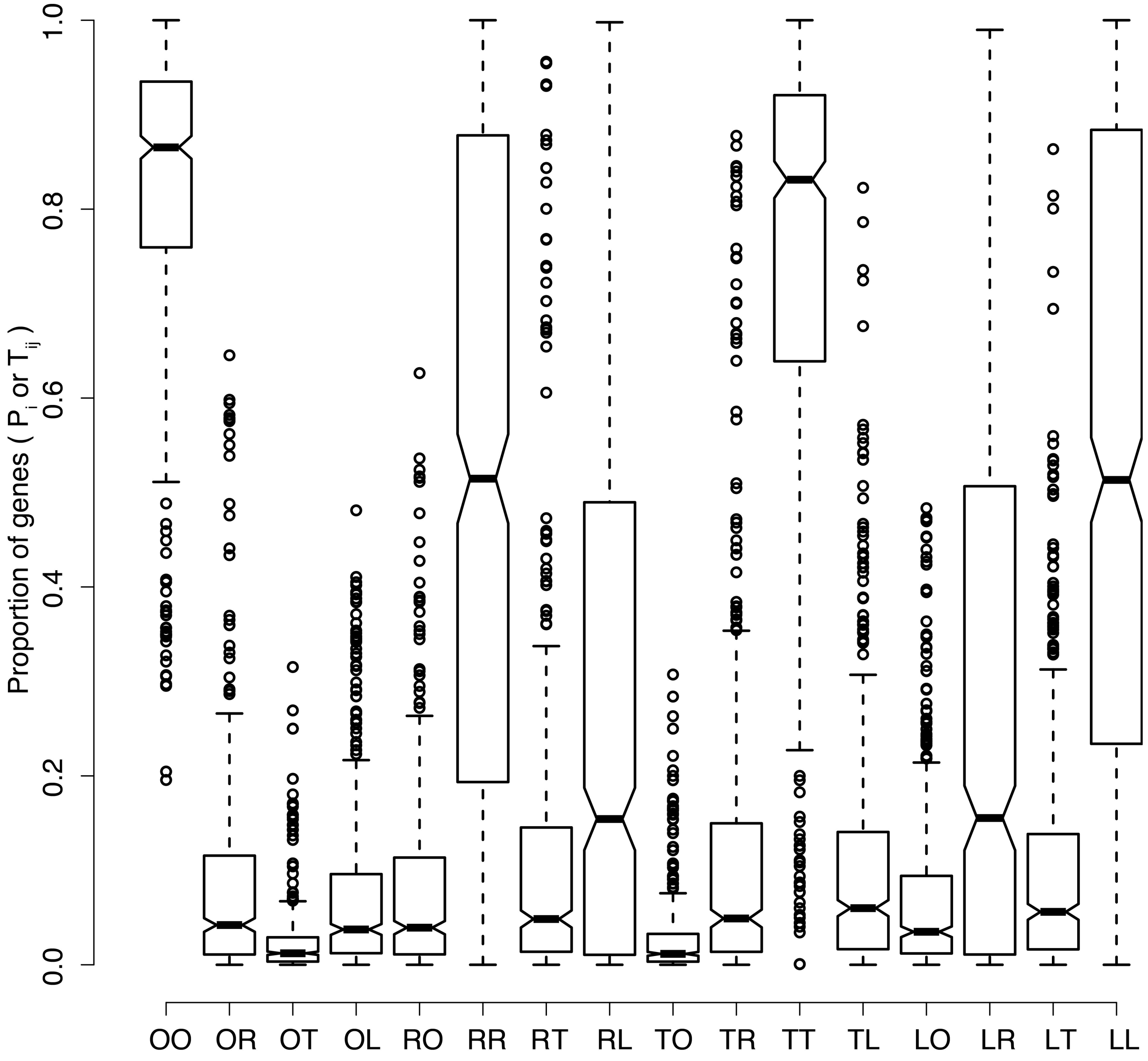

Figure S10 Boxplot representing the proportion of genes translocating to the same bin and across different chromosomal bins (O, T, R and L). For example genes conserved in the Origin bin are denoted as OO and genes translocating form the Origin bin to the Right bin are denoted as OR and so on.
