# Supplementary material for: Comparative Genomics of Interreplichore Translocations in Bacteria: A Measure of Chromosome Topology?
Source: G3 (Bethesda). 2016 Mar 30;6(6):1597–606. doi: 10.1534/g3.116.028274 (PMC4889656; doi:10.1534/g3.116.028274)
Supplement: Supplemental Material [file supp_g3.116.028274_FigureS11.pdf]

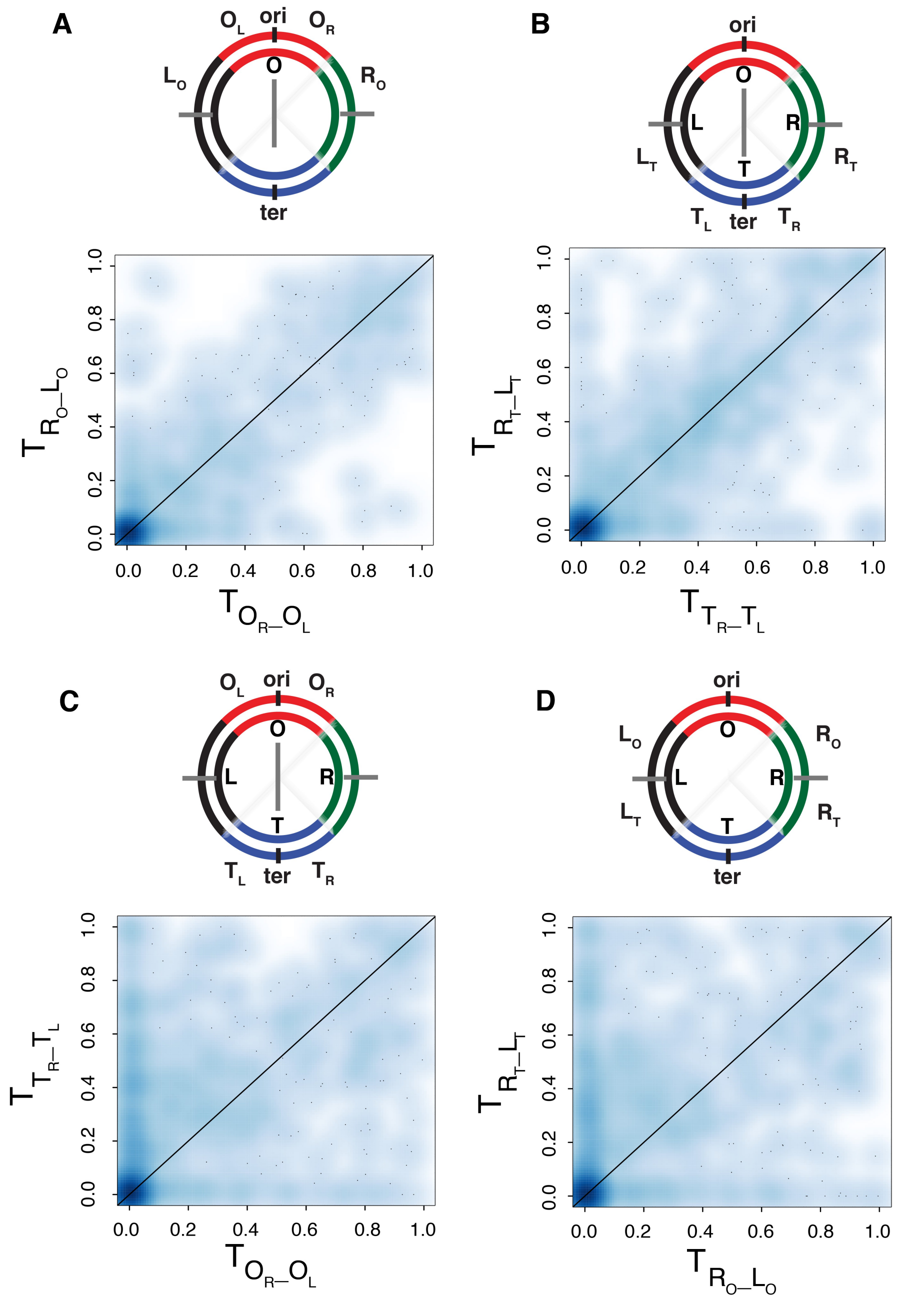

Figure S11 A) Plot representing the proportion of inter-replichore translocations between R_O and L_O (as per schematic) versus translocations within the O bin, but across replichores (O_R and O_L) ($\rho_{Spearman}$ = 0.8, *P*-value < 10^{-10}). The intensity of blue color represents density of points on the scatter plot. The black line has an intercept at 0 and slope of 1 (the color coding is similar across all four figures A, B, C and D); B) Plot representing the proportion of inter-replichore translocations between R_T and L_T (as per schematic) versus those within the T bin (T_R and T_L) ($\rho_{Spearman}$ = 0.72, *P*-value < 10^{-10}); C) Plot showing the relatively higher proportion of inter-replichore translocations within the T bin (T_R and T_L) as compared to within the O bin (O_R and O_L) (*P*-value < 10^{-8}, Wilcoxon test); D) Plot showing the relatively higher proportion of inter-replichore translocations R_T and L_T as compared to R_O and L_O (*P*-value < 10^{-7}, Wilcoxon test).
