# Supplementary material for: Comparative Genomics of Interreplichore Translocations in Bacteria: A Measure of Chromosome Topology?
Source: G3 (Bethesda). 2016 Mar 30;6(6):1597–606. doi: 10.1534/g3.116.028274 (PMC4889656; doi:10.1534/g3.116.028274)
Supplement: Supplemental Material [file supp_g3.116.028274_FigureS13.pdf]

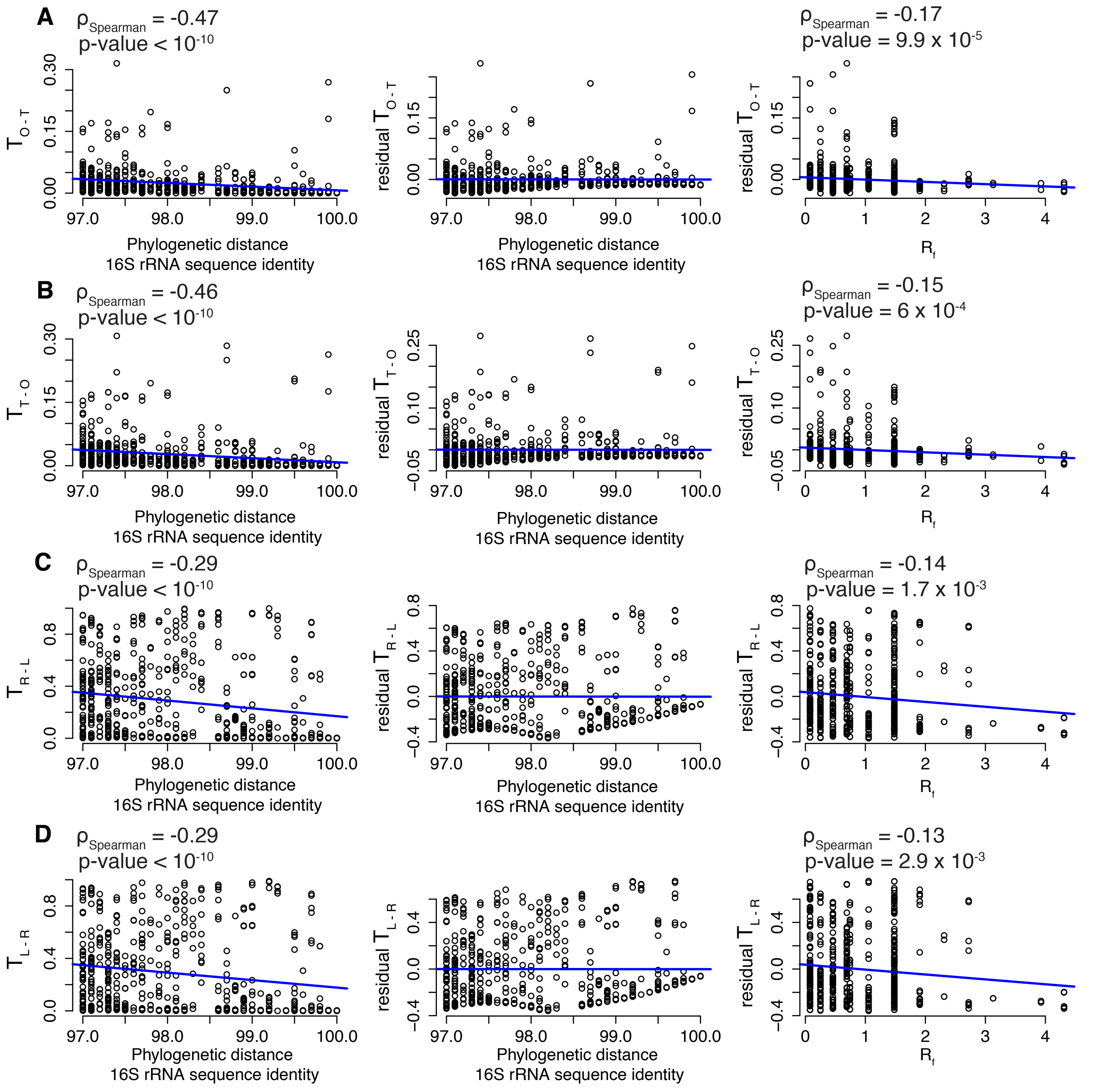

Figure S13 (A, left) Plot representing the dependence of $T_{O\text{-}T}$ on phylogenetic distance; (A, middle) Plot representing the absence of dependence of residual $T_{O\text{-}T}$ on phylogenetic distance after correcting for phylogenetic distance; (A, right) Plot representing the dependence of residual $T_{O\text{-}T}$ on R_f. Panel B, C and D represent same data as in Panel A but for $T_{T\text{-}O}$, $T_{R\text{-}L}$ and $T_{L\text{-}R}$ respectively.
