# Supplementary material for: Comparative Genomics of Interreplichore Translocations in Bacteria: A Measure of Chromosome Topology?
Source: G3 (Bethesda). 2016 Mar 30;6(6):1597–606. doi: 10.1534/g3.116.028274 (PMC4889656; doi:10.1534/g3.116.028274)
Supplement: Supplemental Material [file supp_g3.116.028274_FigureS14.pdf]

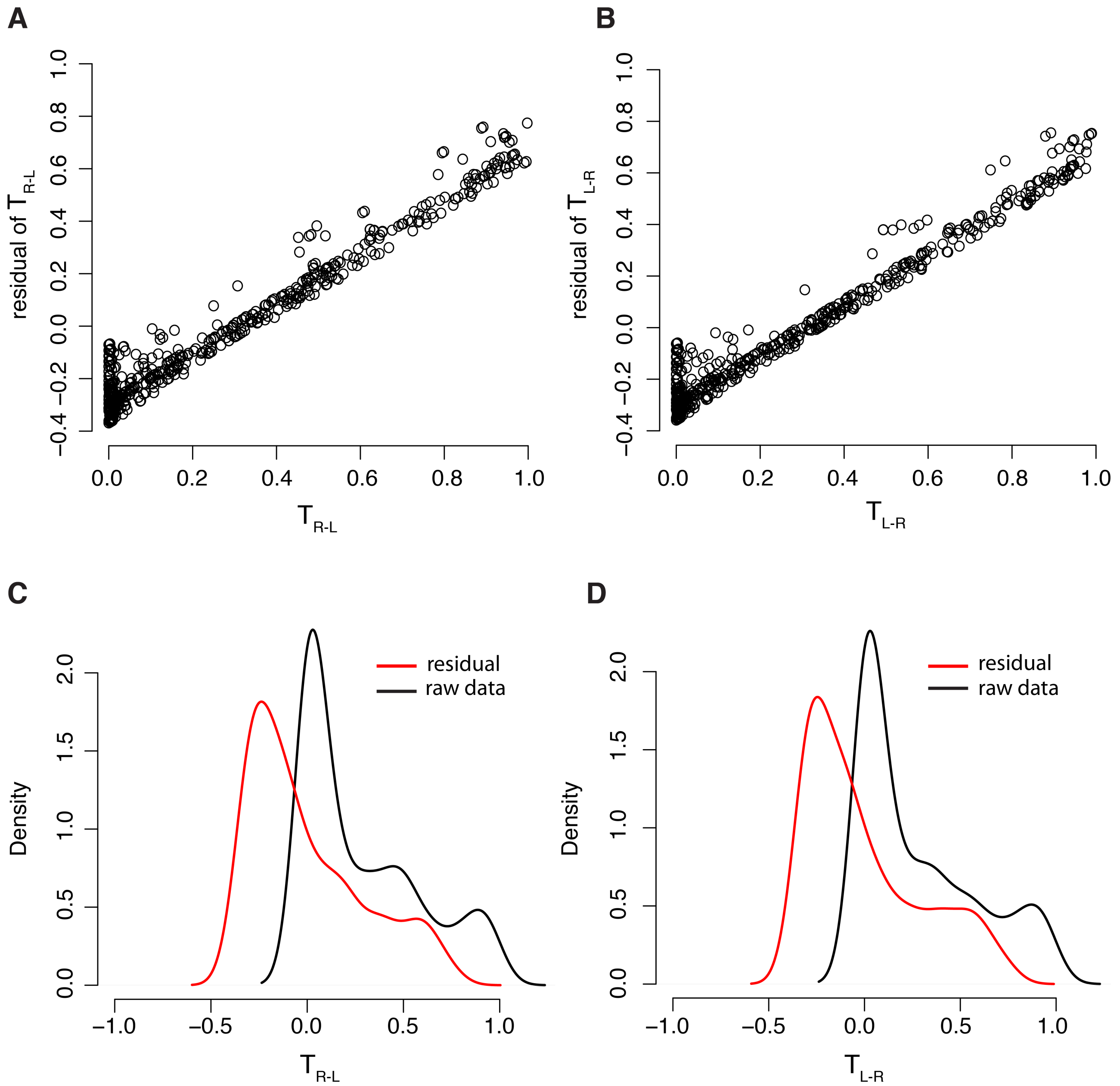

Figure S14 A) Plot representing the relationship between T_{R-L} and the residual of T_{R-L} after correcting for its dependence on phylogenetic distance; B) Plot representing the relationship between T_{L-R} and the residual of T_{L-R} after correcting for its dependence on phylogenetic distance; C) Density plot representing the distribution of T_{R-L} (black) and residual T_{R-L} translocations (red) respectively. D) Density plot representing the distribution of T_{L-R} (black) and residual T_{L-R} translocations (red) respectively.
