# Supplementary material for: Comparative Genomics of Interreplichore Translocations in Bacteria: A Measure of Chromosome Topology?
Source: G3 (Bethesda). 2016 Mar 30;6(6):1597–606. doi: 10.1534/g3.116.028274 (PMC4889656; doi:10.1534/g3.116.028274)
Supplement: Supplemental Material [file supp_g3.116.028274_FigureS15.pdf]

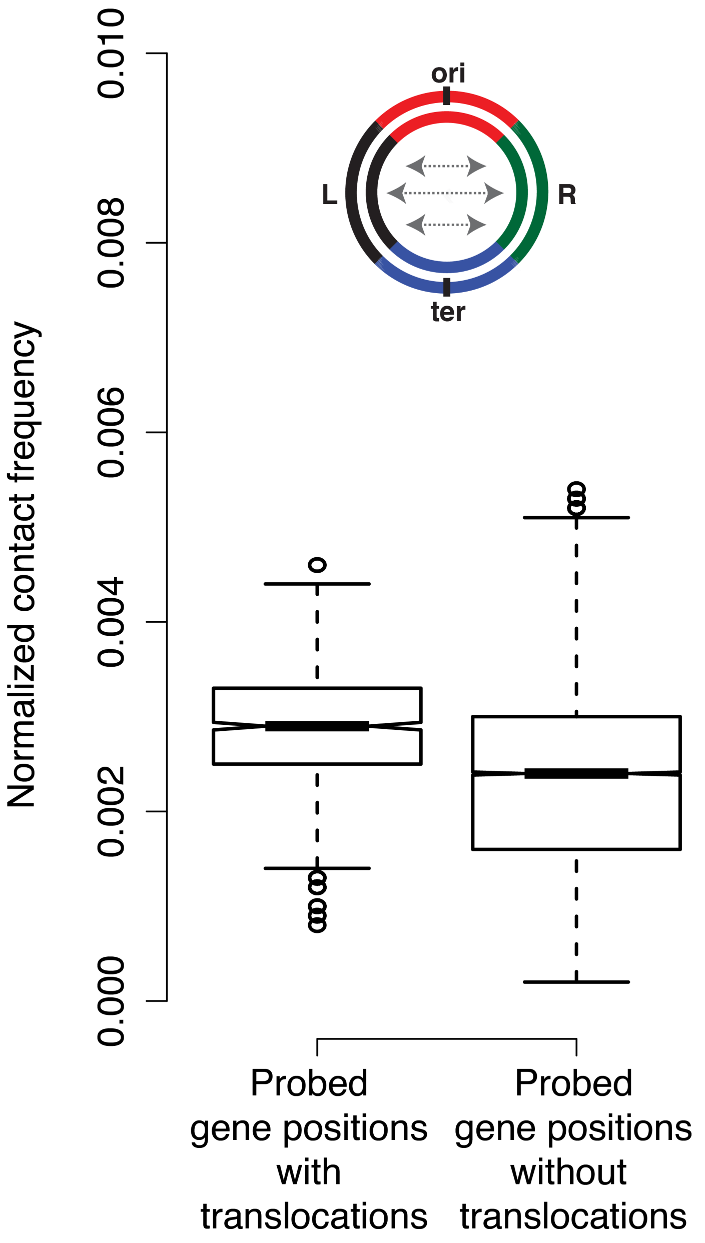

Figure S15 Boxplot representing the normalized inter-replichore contact frequencies between R and L bins as derived from (Le *et al.* 2013) for all probed gene positions with and without inter-replichore translocations in *Caulobacter crescentus* NA1000 (NC_011916) (P-value < 10^{-10}, Wilcoxon test).
