# Supplementary material for: Comparative Genomics of Interreplichore Translocations in Bacteria: A Measure of Chromosome Topology?
Source: G3 (Bethesda). 2016 Mar 30;6(6):1597–606. doi: 10.1534/g3.116.028274 (PMC4889656; doi:10.1534/g3.116.028274)
Supplement: Supplemental Material [file supp_g3.116.028274_FigureS16.pdf]

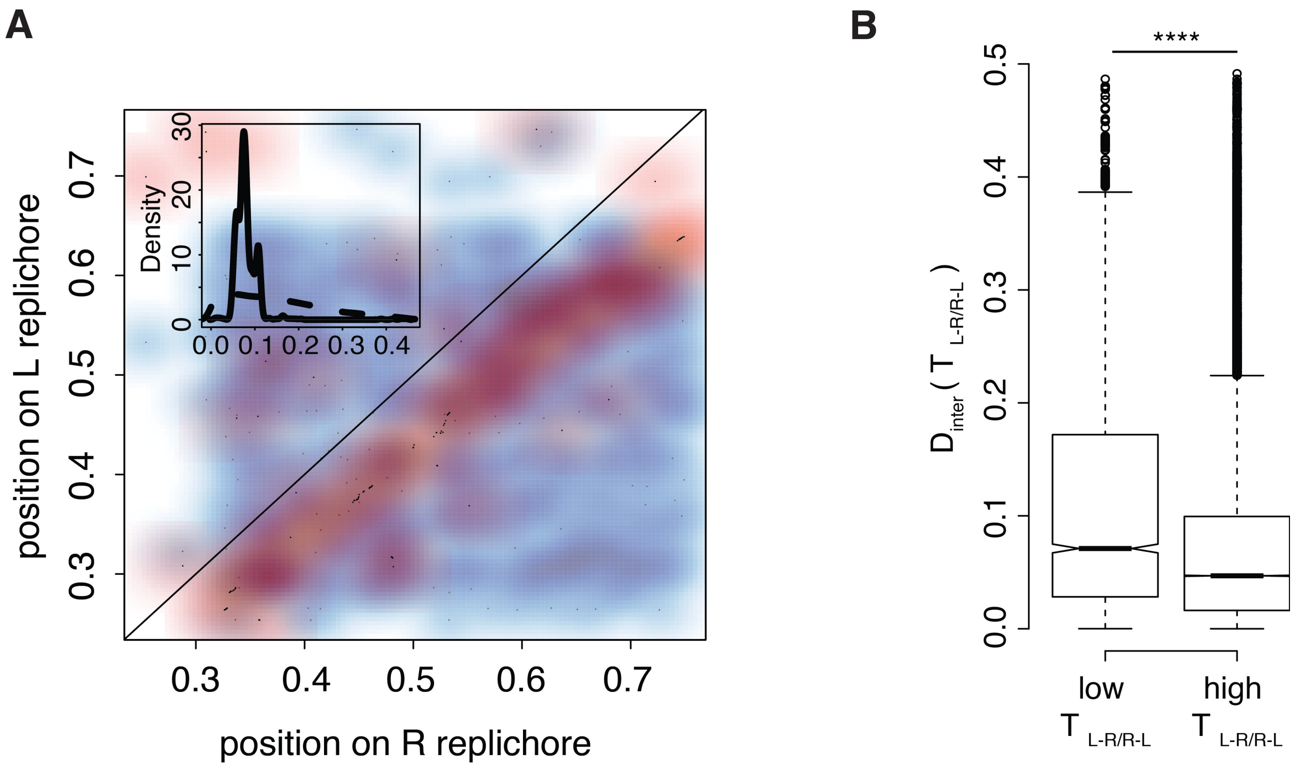

Figure S16 A) Scatterplot representing 727 inter-replichore translocations in red between *Mycobacterium sp.* (NC_014814) and *Mycobacterium smegmatis* (NC_008596). Blue represents a randomized dataset (100 iterations). Inner panel showing the distribution of $D_{inter} = |d_{R/L} - d_{L/R}|$ (solid line) for inter-replichore translocations between *Mycobacterium sp.* (NC_014814) and *Mycobacterium smegmatis* (NC_008596). Dashed line indicates the distribution of D_{inter} for randomized data (as described in methods); B) Boxplot representing the distribution of D_{inter} for bacteria showing low and high inter-replichore translocations (P-value < 10^{-3}, Wilcoxon test). Asterisks indicate p-value < 10^{-3}.
