# Supplementary material for: Comparative Genomics of Interreplichore Translocations in Bacteria: A Measure of Chromosome Topology?
Source: G3 (Bethesda). 2016 Mar 30;6(6):1597–606. doi: 10.1534/g3.116.028274 (PMC4889656; doi:10.1534/g3.116.028274)
Supplement: Supplemental Material [file supp_g3.116.028274_FigureS17.pdf]

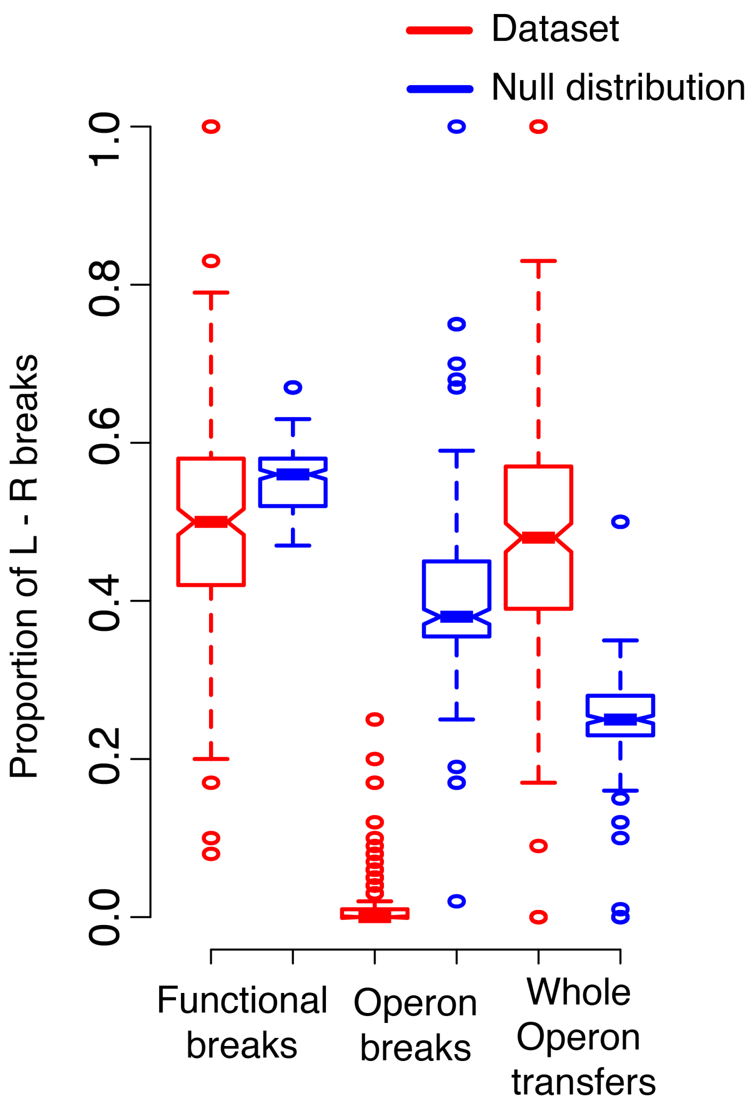

Figure S17 Boxplot representing inter-replichore translocations leading to three kinds of breakpoints 1) functional breaks - breaks in contiguous genes which are functionally similar; 2) Operon breaks – breaks disrupting contiguity of genes belonging to an operon and 3) Whole Operon transfers – breaks that do not disrupt the contiguity of genes belonging to an operon.
