# Supplementary material for: Comparative Genomics of Interreplichore Translocations in Bacteria: A Measure of Chromosome Topology?
Source: G3 (Bethesda). 2016 Mar 30;6(6):1597–606. doi: 10.1534/g3.116.028274 (PMC4889656; doi:10.1534/g3.116.028274)
Supplement: Supplemental Material [file supp_g3.116.028274_FigureS18.pdf]

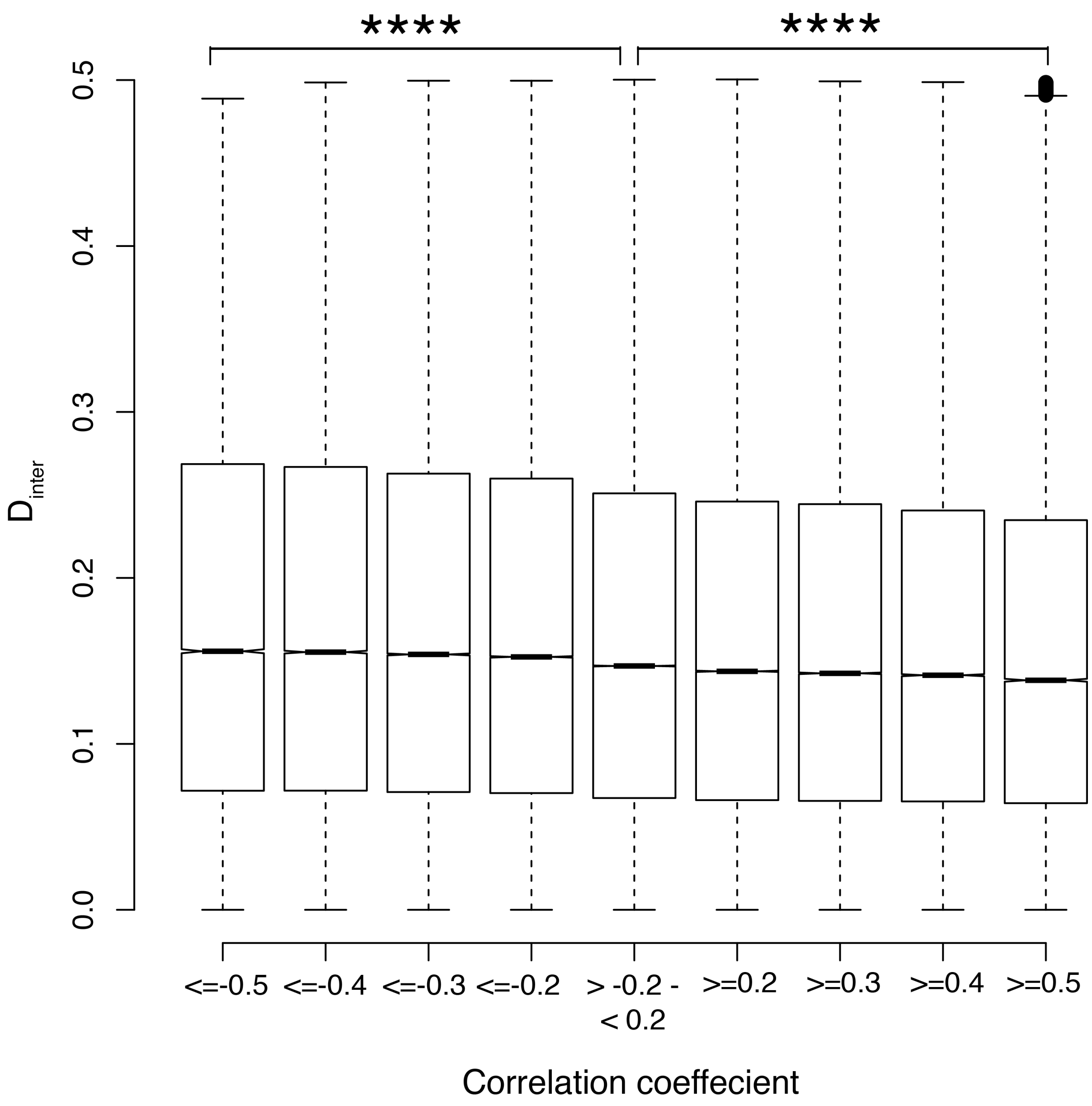

Figure S18 Boxplot representing the distribution of D_{inter} (difference in distance of genes present on two different replichores from *oriC*) in *Shewanella oneidensis* MR1 for different values of correlation in gene expression levels. Weak but statistically significant differences in D_{inter} are observed between gene pairs with high correlation coefficients (>=0.5) and those with low correlation (>-0.2 and <0.2). (*P*-value < 10^{-10}, Wilcoxon test). Similarly weak but statistically significant differences in D_{inter} are observed between gene pairs with high negative correlation coefficients (<=-0.5) and those with low correlation (>-0.2 and <0.2). (*P*-value < 10^{-10}, Wilcoxon test). Asterisks indicate p-value < 10^{-3}.
