# Supplementary material for: Comparative Genomics of Interreplichore Translocations in Bacteria: A Measure of Chromosome Topology?
Source: G3 (Bethesda). 2016 Mar 30;6(6):1597–606. doi: 10.1534/g3.116.028274 (PMC4889656; doi:10.1534/g3.116.028274)
Supplement: Supplemental Material [file supp_g3.116.028274_FigureS19.pdf]

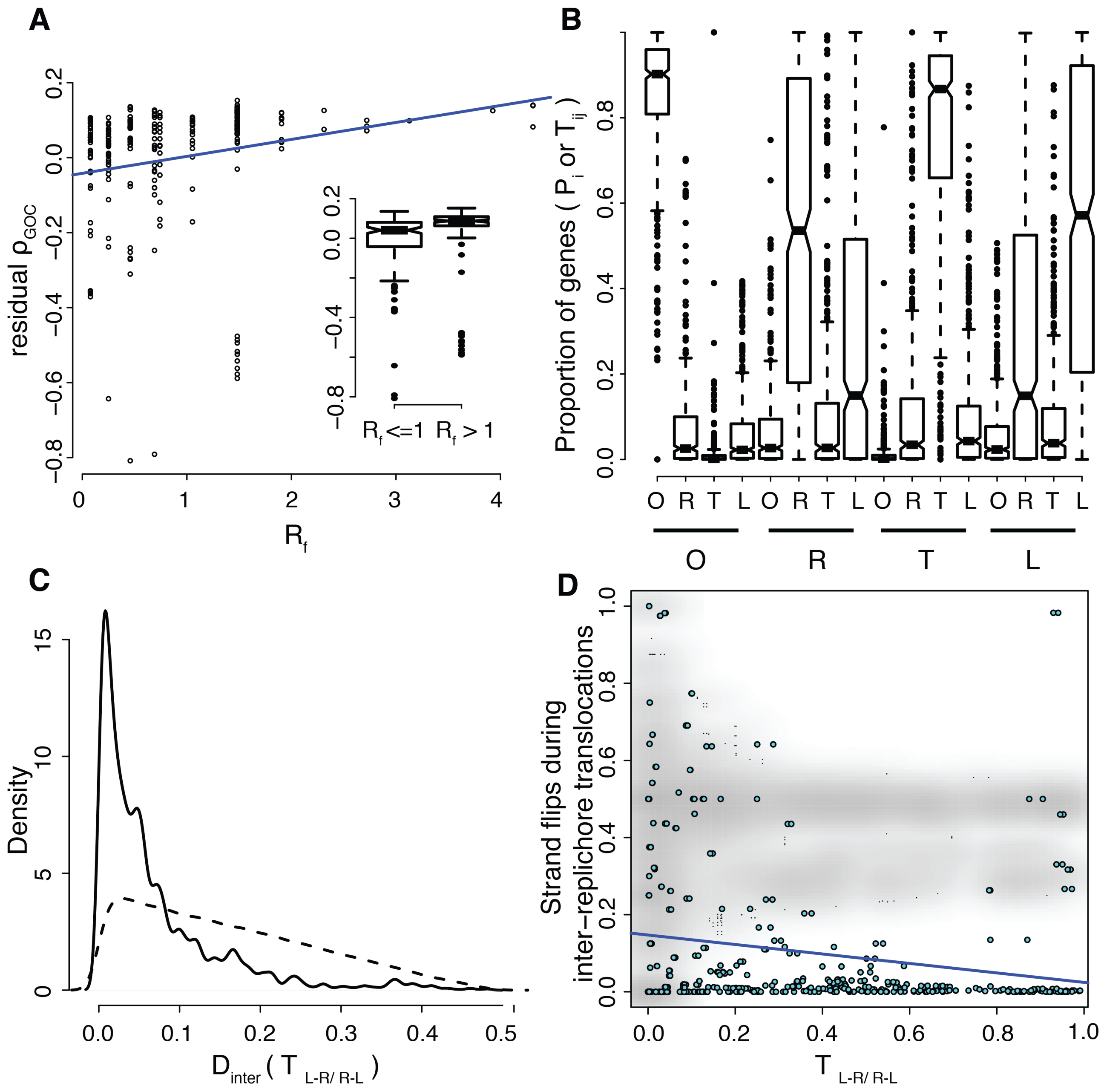

Figure S19 To make the selection of orthologs more stringent, we selected a subset of orthologs that showed >=80% sequence identity. We repeated the key analyses described in results using this subset alone. A) Plot representing the correlation between residual Gene Order Conservation and R_f ($\rho_{Spearman}$ = 0.37, *P*-value < 10^{-10}). Inner panel showing statistically significant difference between residual GOC for slow (R_f <= 1) and fast (R_f > 1) growing bacteria (*P*-value = 1.1 x 10^{-9}, Wilcoxon test); B) Boxplot representing the proportion of genes translocating to the same bin and to different chromosomal bins; C) Plot similar to **Figure 5A** representing the distribution of $D_{inter} = |d_{Ri/Lj} - d_{Li/Rj}|$ for all orthologs showing >=80% sequence identity. The dashed line depicts the null distribution derived from our randomization control, and the solid line indicates the actual data; D) Plot representing the proportion of leading-lagging strand flips post inter-replichore translocations as a function of the proportion of inter-replichore translocations ($\rho_{Spearman}$ = 0.14, *P*-value = 0.001) in cyan and the randomized dataset (50 iterations) in grey.
