# Supplementary material for: Comparative Genomics of Interreplichore Translocations in Bacteria: A Measure of Chromosome Topology?
Source: G3 (Bethesda). 2016 Mar 30;6(6):1597–606. doi: 10.1534/g3.116.028274 (PMC4889656; doi:10.1534/g3.116.028274)
Supplement: Supplemental Material [file supp_g3.116.028274_FigureS20.pdf]

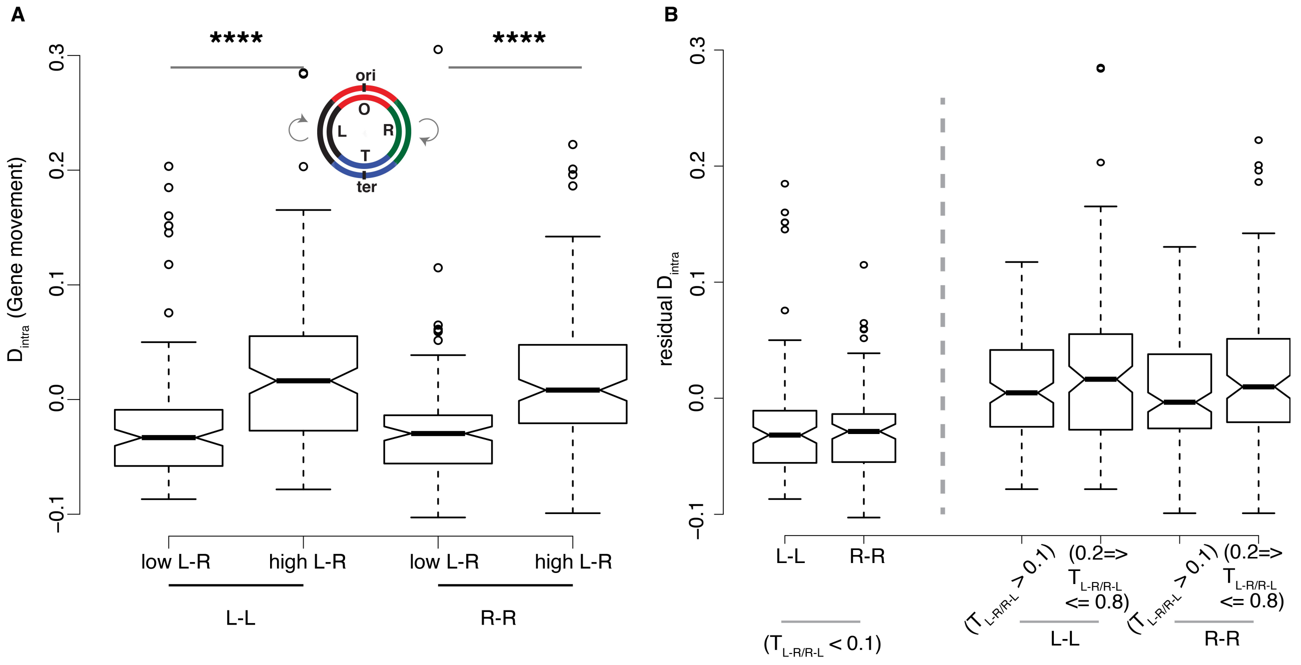

Figure S20 A) Boxplot representing gene movement as residual (D_{intra}) [D_{intra} is the difference (D_{intra}= $|d_{Ri} - d_{Rj}|$ and $|d_{Li} - d_{Lj}|$) in positions of orthologous pairs of genes in genomes *i* and *j* where both members of the pair are encoded on the same replichore] after correcting for the dependence of D_{intra} on phylogenetic distance. For genes conserved in R bin (R-R, P-value < 10^{-10}, Wilcoxon test) and L bin (L-L, P-value < 10^{-10} , Wilcoxon test) for bacteria with high and low inter-replichore translocations. Asterisks indicate p-value < 10^{-3}; B) Boxplot representing residual D_{intra} for $T_{L-R/R-L}$ > 0.1 and 0.2 >= $T_{L-R/R-L}$ <= 0.8 for both the left (LL, *P*-value =0.3, Wilcoxon test) and the right replichore (RR, *P*-value = 0.1, Wilcoxon test). (This analysis was done to test whether our conclusions are a possible artifact of problems in the annotation of circular genomes, wherein a replichore defined as R in our study is, in fact, L and vice-versa, we performed the following control. Our analysis primarily deals with a binary classification of genome pairs into those showing high or low $T_{L-R/R-L}$, and does not depend on the exact value of $T_{L-R/R-L}$. The wrong assignment of replichores for one member of a genome pair will affect our results only when $T_{L-R/R-L}$ is extremely high. We removed all genome pairs with $T_{L-R/R-L}$ > 0.8 from the list of genomes with high $T_{L-R/R-L}$ and found that D_{intra} is not significantly different for this dataset.)
