# Supplementary material for: Comparative Genomics of Interreplichore Translocations in Bacteria: A Measure of Chromosome Topology?
Source: G3 (Bethesda). 2016 Mar 30;6(6):1597–606. doi: 10.1534/g3.116.028274 (PMC4889656; doi:10.1534/g3.116.028274)
Supplement: Supplemental Material [file supp_g3.116.028274_FigureS21.pdf]

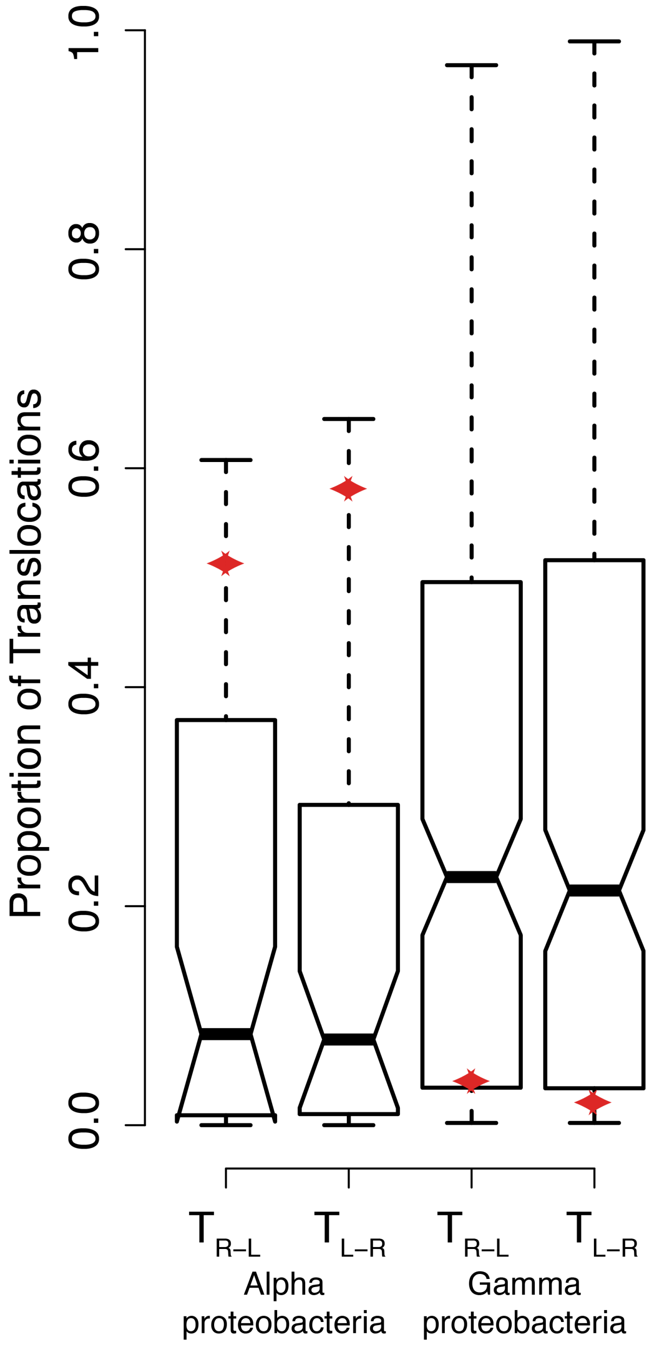

Figure S21 Boxplot representing the distribution of $T_{L\text{-}R/R\text{-}L}$ in Alpha and Gamma proteobacteria. $T_{L\text{-}R/R\text{-}L}$ values for *Caulobacter crescentus* are marked in red under Alpha-proteobacteria. $T_{L\text{-}R/R\text{-}L}$ values for *E. coli* are marked in red under Gamma-proteobacteria.
