# Supplementary material for: Comparative Genomics of Interreplichore Translocations in Bacteria: A Measure of Chromosome Topology?
Source: G3 (Bethesda). 2016 Mar 30;6(6):1597–606. doi: 10.1534/g3.116.028274 (PMC4889656; doi:10.1534/g3.116.028274)
Supplement: Supplemental Material [file supp_g3.116.028274_FigureS22.pdf]

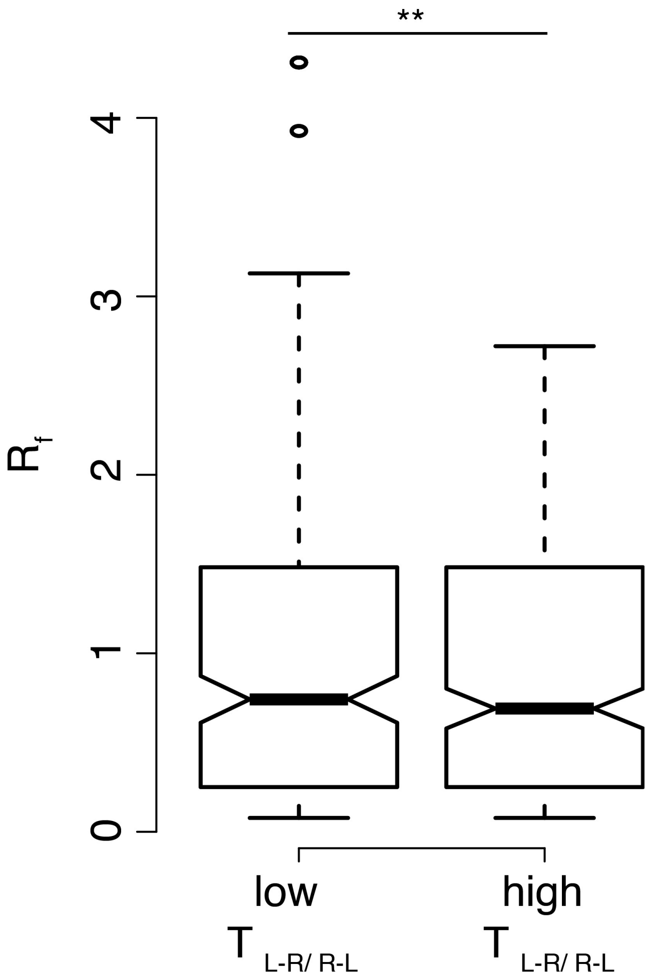

Figure S22 Boxplot representing the distribution of R_f for bacteria with low and high inter-replichore translocations (*P*-value = 0.03, Wilcoxon test).
