# Supplementary material for: Comparative Genomics of Interreplichore Translocations in Bacteria: A Measure of Chromosome Topology?
Source: G3 (Bethesda). 2016 Mar 30;6(6):1597–606. doi: 10.1534/g3.116.028274 (PMC4889656; doi:10.1534/g3.116.028274)
Supplement: Supplemental Material [file supp_g3.116.028274_FileS1.pdf]

COG categories

INFORMATION STORAGE AND PROCESSING

[J] Translation, ribosomal structure and biogenesis; [A] RNA processing and modification; [K] Transcription; [L] Replication, recombination and repair; [B] Chromatin structure and dynamics.

CELLULAR PROCESSES AND SIGNALING

[D] Cell cycle control, cell division, chromosome partitioning; [Y] Nuclear structure; [V] Defense mechanisms; [T] Signal transduction mechanisms; [M] Cell wall/membrane/envelope biogenesis; [N] Cell motility; [Z] Cytoskeleton; [W] Extracellular structures; [U] Intracellular trafficking, secretion, and vesicular transport; [O] Posttranslational modification, protein turnover, chaperones.

METABOLISM

[C] Energy production and conversion; [G] Carbohydrate transport and metabolism; [E] Amino acid transport and metabolism; [F] Nucleotide transport and metabolism; [H] Coenzyme transport and metabolism; [I] Lipid transport and metabolism; [P] Inorganic ion transport and metabolism; [Q] Secondary metabolites biosynthesis, transport and catabolism.

POORLY CHARACTERIZED

[R] General function prediction only and Function unknown.
