# Supplementary material for: Comparative Genomics of Interreplichore Translocations in Bacteria: A Measure of Chromosome Topology?
Source: G3 (Bethesda). 2016 Mar 30;6(6):1597–606. doi: 10.1534/g3.116.028274 (PMC4889656; doi:10.1534/g3.116.028274)
Supplement: Supplemental Material [file supp_g3.116.028274_FileS2.pdf]

References

Delcher, A. L., D. Harmon, S. Kasif, O. White, and S. L. Salzberg, 1999 Improved microbial gene identification with GLIMMER. Nucleic Acids Res. 27: 4636–4641.

Edgar, R. C., 2004 MUSCLE: multiple sequence alignment with high accuracy and high throughput. Nucleic Acids Res. 32: 1792–1797.

Freilich, S., A. Kreimer, E. Borenstein, N. Yosef, R. Sharan *et al.*, 2009 Metabolic-network-driven analysis of bacterial ecological strategies. Genome Biol. 10: R61.

Le, T. B. K., M. V. Imakaev, L. A. Mirny, and M. T. Laub, 2013 High-resolution mapping of the spatial organization of a bacterial chromosome. Science 342: 731–734.

Tamura, K., and M. Nei, 1993 Estimation of the number of nucleotide substitutions in the control region of mitochondrial DNA in humans and chimpanzees. Mol. Biol. Evol. 10: 512–526.

Vieira-Silva, S., and E. P. C. Rocha, 2010 The Systemic Imprint of Growth and Its Uses in Ecological (Meta)Genomics (N. A. Moran, Ed.). PLoS Genet. 6: e1000808.
